# Closed-loop real-time simulation model of hemodynamics and oxygen transport in the cardiovascular system

**DOI:** 10.1186/1475-925X-12-69

**Published:** 2013-07-10

**Authors:** Michael Broomé, Elira Maksuti, Anna Bjällmark, Björn Frenckner, Birgitta Janerot-Sjöberg

**Affiliations:** 1 ECMO Department, Karolinska University Hospital, Stockholm, SE-171 76, Sweden; 2 Anesthesiology and Intensive Care, Department of Physiology and Pharmacology, Karolinska Institutet, Stockholm, Sweden; 3 School of Technology and Health, KTH Royal Institute of Technology, Stockholm, Sweden; 4 Department of Molecular Medicine and Surgery, Karolinska Institutet, Stockholm, Sweden; 5 Department of Pediatric Surgery, Karolinska University Hospital, Stockholm, Sweden; 6 Department of Clinical Science, Intervention and Technology, Karolinska Institutet, Stockholm, Sweden; 7 Department of Clinical Physiology, Karolinska University Hospital, Stockholm, Sweden

**Keywords:** Cardiovascular simulation, Time-varying elastance functions, Lumped parameter model, Valve model, Oxygen transport model, Computer simulation

## Abstract

**Background:**

Computer technology enables realistic simulation of cardiovascular physiology. The increasing number of clinical surgical and medical treatment options imposes a need for better understanding of patient-specific pathology and outcome prediction.

**Methods:**

A distributed lumped parameter real-time closed-loop model with 26 vascular segments, cardiac modelling with time-varying elastance functions and gradually opening and closing valves, the pericardium, intrathoracic pressure, the atrial and ventricular septum, various pathological states and including oxygen transport has been developed.

**Results:**

Model output is pressure, volume, flow and oxygen saturation from every cardiac and vascular compartment. The model produces relevant clinical output and validation of quantitative data in normal physiology and qualitative directions in simulation of pathological states show good agreement with published data.

**Conclusion:**

The results show that it is possible to build a clinically relevant real-time computer simulation model of the normal adult cardiovascular system. It is suggested that understanding qualitative interaction between physiological parameters in health and disease may be improved by using the model, although further model development and validation is needed for quantitative patient-specific outcome prediction.

## Background

The increasing number of surgical and medical treatment options in cardiovascular disease imposes a need for better understanding of patient-specific pathology and outcome prediction. Due to the complexity of the cardiovascular system, it is not always possible to understand the role of every cardiovascular parameter; additionally, prediction of the hemodynamic features can be even more challenging when several of those parameters change simultaneously. In many cases the use of simulation models can improve the understanding of both normal physiology and pathophysiology. Moreover, clinical training in simulation centres, where simulation models have been connected to mannequins responding to therapeutic measures, has proved to further increase diagnostic and therapeutic accuracy [1].

Other authors [2] have published complex hemodynamic models based on a reductionistic approach, where contractions are based on cellular properties including electrophysiology, ion channels, myofilament structure and fiber orientation. These models are relevant for exploring normal and disturbed contractile mechanisms as well as integrated organ function. The computational load in 3D models is however presently too large to allow clinical and educational use in studies of central hemodynamics. Simpler models based on cellular properties [3] tend to be less relevant on higher explanatory levels when studying integrated properties of the entire cardiovascular system, although being computationally less demanding.

Models focusing on particular aspects of hemodynamics such as mitral valve dynamics [4], remodeling of heart and vessels [5], individual drug effects [6] and effects of therapeutic measures such as positive end-expiratory pressure (PEEP) [7] offer good examples of how cardiovascular simulation models can be validated in specific situations and used clinically. It is however difficult to know to what extent these models can be used in clinical situations other than those described in the articles.

The aim of this paper is to present a closed-loop, real-time, lumped parameter cardiovascular simulation model, which includes the behavior of the four cardiac chambers, interatrial and interventricular septum, pericardium, intrathoracic pressure changes, heart valves, intracardiac shunts and vascular system. The current model is based on previous publications [8,9]. The circulatory system interacts with pericardial as well as intrathoracic pressures as in one of the previous models [9] and several improvements have been made. The vascular system has been expanded and the circulatory system closed in order to reach more realistic steady state output when parameters in the model are changed. The systolic cardiac model is replaced in order to more realistically simulate cardiac work and valvular timing. Passive diastolic properties are now non-linear [10] resulting in more realistic changes of filling pressures with ventricular dilatation. Atrial septal interactions are included in addition to a modified ventricular septum [9]. The valves are gradually opening and closing [11] and adjustment of valvular properties therefore allow more realistic simulation of both stenotic and regurgitant valves. Potential atrial septal defects, ventricular septal defects and persistent ductus arteriosus have been included. An oxygen transport model allowing studies of oxygen delivery and uptake including myocardial oxygen balance have been developed. The most unique feature of this model is however not any particular advanced modeling solutions in each sub-part, but rather the holistic approach giving a comprehensive overview of cardiovascular hemodynamics in health and disease. The possibility to change the model parameters one by one facilitate understanding in educational sessions, but real-time simulation and fast feed-back after parameter changes are also important features in a clinical decision support scenario in cardiac surgery and intensive care.

The model is presented from a normal cardiovascular physiological point of view, but a limited number of pathological scenarios will also be shown. A secondary aim is to show that the model generates valid output, which is consistent with earlier published clinical and experimental data, focusing on the behavior of the entire system rather than on single variables.

## Methods

### General overview of the model

**Figure 1:**
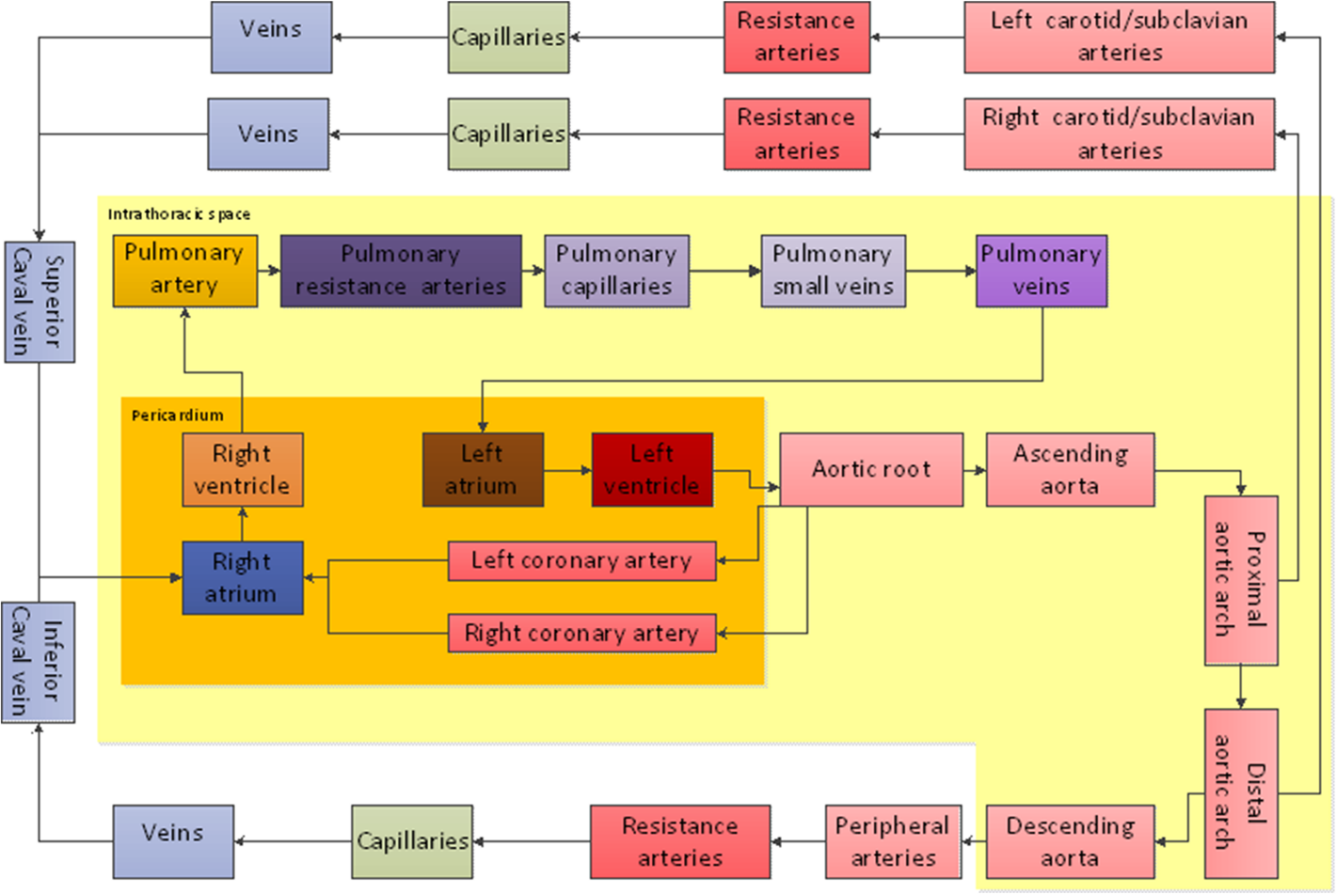
**Sketch showing the cardiac and vascular components of the simulation model.** The dark yellow area is the pericardium containing the cardiac chambers and coronary vessels. The light yellow area is the intra-thoracic space containing the pericardium, the pulmonary circulation and the thoracic aorta. The extra-thoracic space contains the carotid/subclavian circulation in the upper part and the rest of the systemic circulation in the lower left part.

The model (Figure 1) is based on the following compartments: the four cardiac chambers and corresponding valves, the pericardium, the systemic circulation consisting of the aortic root, ascending aorta, proximal aortic arch, right carotid/subclavian artery, distal aortic arch, left carotid/subclavian artery, descending aorta and the representation of a peripheral arterial system (See also electrical analogue in Additional file 1: Figure S1). The peripheral arterial system contains resistance arteries (arteriolae), capillaries and small veins emptying into the inferior caval vein, while the two carotid/subclavian arteries in a similar way end up in the superior caval vein. The pulmonary circulation is represented by the main pulmonary trunk, pulmonary resistance arteries (arteriolae), capillaries, small veins and pulmonary veins emptying into the left atrium. Two coronary vessels are also included. Input to the vascular model are physical dimensions [12,13], tissue properties and number of parallel vessels in each compartment modified to fit the anatomical structure of the present vascular model; while pressure, flow, volume and oxygen saturation are simulated outputs and can be displayed. The cardiac, valvular, pericardial and vascular properties can be modified in detail by changing the parameters presented in the supplement (Additional file 2: Table S1-5). Model complexity is chosen to enable future simulations of central hemodynamics in major congenital and acquired circulatory pathologies and extra-corporeal circulatory support.

### Cardiac model

#### The heart chambers

**Figure 2:**
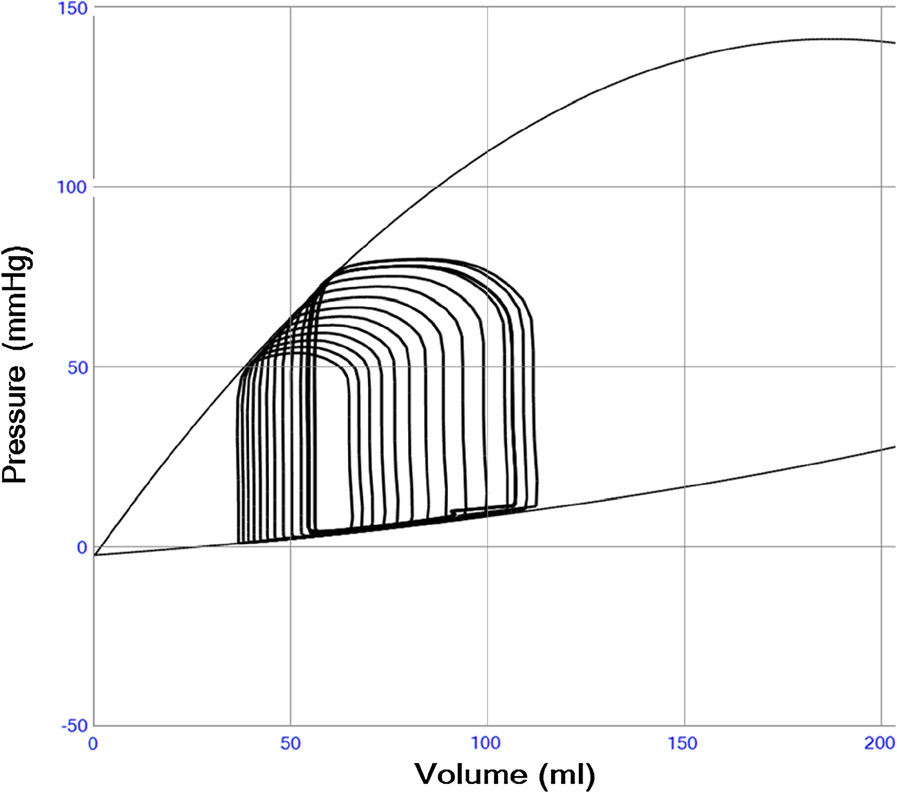
**Left ventricular pressure-volume loops during a preload reduction maneuver in a case with slightly reduced left ventricular function (E**_**max **_**1.4 and E**_**min **_**0.08).** The diastolic and end-systolic pressure-volume relations are shown in thin black lines. The end-systolic line is curved due to the “heart law of Starling” reducing contractile strength during volume overload according to Additional file 2: Equation S2. The curved end-diastolic relation illustrates the increase in passive stiffness when the ventricle is dilated according to Additional file 2: Equation S3.

The four cardiac chambers passive and active properties are modeled with separate time-varying elastance functions based on the “Double-Hill equation” as previously described [11,14] including a slightly modified Starling mechanism (Figure 2) [8]. The model includes internal chamber flow resistance and viscous wall properties of the heart, in accordance with previous models [9,11]. Details are shown in the cardiac model section of the supplement.

By using a continuous function through the cardiac cycle manual setting of valvular timing [9] is avoided. Each cardiac chamber has its own elastance function of realistic shape (Additional file 3: Figure S3). Timing and duration of contractions in the cardiac chambers can be set independently, in order to create variable atrioventricular (AV) and interventricular (VV) intervals as well as realistic timing of valve opening and closure. Thereby the model offers additional opportunities to simulate arrhythmias as well as interventricular dyssynchronicity. As an example atrial fibrillation can be simulated by turning off atrial contractions and randomizing the time between ventricular contractions.

The electrocardiogram (ECG) (Additional file 4: Figure S4; Additional file 5: Figure S5) is simulated by summing the four cardiac elastance functions with a linear weight factor, based on the approximate distance from each cardiac chamber to the chosen surface ECG lead assuming an electromechanical delay of 60 ms. The ECG does not by itself influence the simulated physiology and is included as an output of the model only to add clinical realism during educational sessions, therefore the detailed algorithms are not described.

#### The heart valves

Valve pressure gradients are composed of a Bernoulli resistance and an inertial term (Additional file 2: Equation S4 and Figure S6 of Additional file 6) as earlier described [8,9]. Minimum and maximum valvular areas can be set to simulate valvular stenosis and regurgitation. The valve areas change gradually (Additional file 4: Figure S4) as a function of pressure gradients and valve inertial constants [11] as is shown in the supplement (Additional file 2: Table S4). Blood flow inertance ***L*** is determined by density ***ρ***, the variable inflow length ***l*** and the valve area ***A*** (Equation 1). Inflow length ***l*** is set to a value identical to the instantaneous diameter of the valve in contrast to the constant value in Mynard *et al.*[11].

(1) $$L=\frac{\rho \cdot l}{A}=\frac{\rho \cdot 2\cdot r}{\pi \cdot r^{2}}=\frac{\rho \cdot 2}{\pi \cdot r}$$

#### The atrial and ventricular septum

The interventricular septal displacement is simulated using pressure transmission through the muscular septum as previously described [9,15] (Equation 2). As a further development the ventricular septal stiffness ***E***_***sv***_ is modeled in order to increase in proportion to the left ventricular systolic elastance ***e***_***lv***_***(t)*** (Equation 3), thereby stiffening during contraction, in accordance with known physiological characteristics. Additional file 5: Figure S5 shows the ventricular septal shift at different stiffness.

(2) $$p_{\mathrm{lv}}=\frac{E_{\mathrm{sv}}}{E_{\mathrm{sv}}+e_{\mathrm{lv}}}\cdot e_{\mathrm{lv}}\cdot v_{\mathrm{lv}}+\frac{e_{\mathrm{lv}}}{E_{\mathrm{sv}}+e_{\mathrm{lv}}}\cdot p_{\mathrm{rv}}$$

(3) $$E_{\mathrm{sv}}=E_{\mathrm{sv}0}\cdot e_{\mathrm{lv}}\left(t\right)$$

Atrial septal interaction is modeled in a similar way. The septal interaction does not give any significant contribution during normal physiology in apnea, but is important when simulating interventricular dyssynchrony, variations in intrathoracic pressures including artificial ventilation and changes in ventricular loading conditions.

#### The pericardium

In order to resemble pericardial function in health and disease a user-defined exponential function relating intra-pericardial pressure to total heart volume is adopted from the literature [9] (Additional file 7: Figure S7). As a further development of the previous model the minimal pericardial pressure is allowed to be a negative value in agreement with what is found experimentally when measured during intrathoracic pressure changes or hypovolemia [16]. Further details can be found in the supplement.

#### Intracardiac and arteriovenous shunts

Atrial septal defects, ventricular septal defects and persistent ductus arteriosus flows are calculated according to Equation 4, with instantaneous flow ***Q*** proportional to the area ***A*** of the defect, the square-root of the pressure difference ***∆P*** and the Gorlin empirical constant ***C*** assumed to be 1 [17]. Acceleration ***g*** due to gravity is set to 980 cm/s^2^.

(4) $$Q=C\cdot A\cdot \sqrt{2\cdot g\cdot \mathrm{\Delta P}}=44.3A\cdot \sqrt{\mathrm{\Delta P}}$$

### Vascular model

#### The systemic circulation

Transmural pressure ***p*** in each vascular compartment is related to the variable segmental vascular volume ***v*** according to an exponential relation (Additional file 2: Equation S7, Additional file 8: Figure S12) [9] creating an increase in stiffness with progressive distension of the vascular wall.

Resistance ***R***_***0***_***,*** inertia ***I***_***0***_***,*** and elastance ***E***_***0***_ at normal mean pressure ***P***_***0***_ are calculated from published and estimated vascular properties [12,13], and relations [18] described in Equations 5–7. These vascular properties are all approximately inversely proportional to ***n***, which is the number of parallel vessels, lumped into each compartment [18]. The parameter ***η*** is blood viscosity, ***l***, ***r***_***0***_ and ***h*** is the length, radius and thickness of a single vessel respectively and ***ρ*** is blood density.

(5) $$R_{0}=\frac{8\cdot \eta \cdot l}{\pi \cdot r_{0}{}^{4}\cdot n}$$

(6) $$I_{0}=\frac{\rho \cdot l}{\pi \cdot r_{0}^{2}\cdot n}$$

(7) $$E_{0}=\frac{Y_{\mathrm{inc}}\cdot h}{2\cdot \pi \cdot r_{0}^{3}\cdot l\cdot n}$$

The same value of Young’s modulus ***Y***_***inc***_ is used in every vascular segment, implying that vascular stiffness properties are solely explained by this constant and vascular dimensions. The actual volume-dependent segmental elastances, resistances and inertias are updated in each calculation step based on the volume and radius assuming constant vessel length and thickness. Details can be found in the blood vessels section of the supplement (Additional file 2: Table S5-7).

The resistance Ω, in series with the capacitor situated in parallel to the blood flow in every vascular segment, corresponds to the viscous property of the vascular wall, dampening the flow pulse. The parameter value assigned to this component is calculated as in Equation 8 with addition of a scaling factor ***λ*** as compared to the characteristic impedance in a classical 3-component Windkessel model [19,20].

(8) $$\Omega =\lambda \cdot \sqrt{L\cdot E}$$

**Figure 3:**
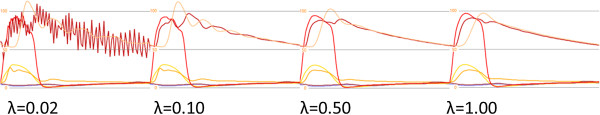
**Effects of changing vascular damping factor λ on cardiac and vascular pressures (peripheral artery (light red), ascending aorta (dark red), left ventricle (red), pulmonary artery (orange), right ventricle (yellow), left atrium (brown), right atrium (blue)) related to the viscous properties of the vascular walls.** Very low values results in numerical instability in the model. A value of 0.5 is chosen in all presented simulations.

The same formula was used to calculate Ω in every vascular segment as previously suggested [19], although the name characteristic impedance is less well suited in a distributed model. A scaling factor λ between 0.0 and 1.0 is used to tune the vascular damping properties. The effects of changing the scaling factor λ can be seen in Figure 3.

The systemic capillaries and veins are described by the same principal vascular model as the arteries although inertial effects are of less importance due to lower pulsatility [21] and a larger cross-sectional area. Every single vessel has high resistance and stiffness; however the large amounts of vessels in parallel lumped into each compartment result in both a low resistance (Equation 5) and a low elastance (Equation 7), corresponding to high compliance and being related to their physiological volume reservoir function. The distribution of the blood volume between arteries, capillaries and veins in the systemic and pulmonary circulation in the model is shown in Additional file 2: Table S7.

#### The pulmonary circulation

The pulmonary artery, resistance vessels, capillaries and veins are modeled as in the systemic circulation. An adjustable pulmonary shunt mimics the circulation without gas exchange due to anatomical anomalies or ventilation-perfusion disturbances as in atelectatic lung tissue.

#### The coronary circulation

Coronary circulation is simulated with a left and right coronary artery emptying in the right atrium as described in the supplement (See also Additional file 9: Figure S8). Myocardial oxygen consumption is calculated according to the pressure-volume area concept of Suga *et al.*[22] on a beat-by-beat basis and estimations of myocardial oxygen balance can therefore be calculated based on both supply and demand.

### Oxygen transport

Blood oxygen content in every compartment is calculated as a constant multiplied by the hemoglobin (***Hb****)*, the oxygen saturation (***Sat****)* and the volume of the compartment (***Volume****)* as in clinical routine and most catheterization laboratories [23] (Equation 9)*.*

(9) C=0.0000136·Hb·Sat·Volume

Oxygen saturation within each compartment is considered homogenous and oxygen transport between compartments is proportional to blood flow, hemoglobin level and saturation. Calculations concerning blood mixing are simplified as effects of blood jets, incomplete mixing and physically dissolved oxygen not are taken into account.

### Baroreceptor reflex

A baroreceptor reflex can be activated in the model that affects heart rate, cardiac contractility (maximum elastance) and arterial vascular resistance as in Sun *et al.*[9] and described in the baroreceptor section of the supplement.

### Simulation

The simulator was compiled as a stand-alone software developed in Visual Basic.NET 2010 (Microsoft Corporation, Redmond, WA, USA) and .NET Framework 4.0 (Microsoft Corporation, Redmond, WA, USA). A time step of 0.25 ms was used to assure stability. The implemented 62 differential equations were solved with an implicit Euler numerical method [24]. Mean values were calculated as running arithmetical means to simplify calculations and conserve memory resources. The program version used was Aplysia CorVascSim 4.4.0.62 (Aplysia Medical AB, Stockholm, Sweden). The model can be run in real-time on a standard personal computer (example: Intel processor Core i3 3.06 GHz, RAM 3 Gb, Graphics ATI Radeon HD4550, Windows 7 32/64-bit).

### Parameter settings in simulations of normal physiology, systolic heart failure, diastolic heart failure, aortic stenosis, aortic regurgitation, a Valsalva maneuver, exercise and progressive arteriosclerosis

All hemodynamic data were presented with zero intrathoracic pressure in order to resemble end-expiratory values. With the basic set of parameters presented in the supplement an adult man weighing 70 kg is described with a blood volume of 5600 ml (80 ml/kg), blood viscosity of 0.00024 mmHg · s and blood density of 1.060 g/cm^3^. Systolic left ventricular heart failure was simulated by decreasing the maximum elastance from 2.8 to 1.0 mmHg/ml. Diastolic left ventricular heart failure was simulated by increasing the basal passive diastolic elastance ***E***_***min***_ from 0.05 to 0.12 mmHg/ml. Aortic stenosis was simulated by decreasing the open aortic valve area from 5.0 to 0.7 cm^2^. Aortic regurgitation was simulated by increasing the closed aortic valve area from 0.0 to 0.2 cm^2^. Simulation of a Valsalva maneuver was accomplished by increasing intrathoracic pressure from 0 to 10 mmHg for approximately 20 seconds with and without baroreceptors activated. All other parameters and settings were identical.

The stepwise simulation of exercise was accomplished by increasing heart rate from 72 to 144 min^-1^, increased left and right ventricular contractility by 50% and increased systemic and pulmonary resistance arterial diameters by 50%. The cardiac elastance parameters ***α***_***1***_ and ***α***_***2***_ were scaled with the square-root of the factor changing the heart rate to preserve a realistic relation between systolic and diastolic times. The vascular damping scaling factor ***λ*** was set to 0.5 in all vascular segments and presented simulations. The oxygen saturation in the pulmonary capillaries was set to 99.4%, corresponding to a normal alveolar pO2 of 13.3 kPa and the pulmonary shunt was set to 10% of cardiac output in all presented simulations. Hemoglobin level was set to 140 g/l and systemic oxygen consumption to 250 ml/min in addition to myocardial oxygen extraction. A Young’s modulus ***Y***_***inc***_ of 3000 mmHg (= 0.40 MPa) similar to the value in Wang *et al.*[12] was used to calculate the vascular elastance ***E***_***0***_ at the vessel-specific normal mean pressure ***P***_***0***_ (see Additional file 2: Table S5) in all simulations above. A progressive arteriosclerotic process was simulated by changing vascular stiffness in all vascular segments through variations in Young’s modulus ***Y***_***inc***_ between 2000 and 6000 mmHg. Note that comparisons with real clinical cases will not be performed due to unknown patient-specific secondary adaptations.

### Sensitivity analysis

The relation between changes in input parameters ***x*** and model output variables ***y*** was studied with a sensitivity analysis during simulation of normal physiology (Additional file 10). Input parameters were increased 10% one by one. Seven model output variables [left ventricular end-systolic pressure (LVESP), right ventricular end-systolic pressure (RVESP), left atrial pressure (LAP), right atrial pressure (RAP), left ventricular stroke work (LVSW), right ventricular stroke work (RVSW) and cardiac output (CO)] focusing on cardiac function were evaluated after 60 seconds assuring a hemodynamic steady-state. Sensitivity ***S*** was calculated according to Equation 10.

(10) $$S=\frac{\mathrm{\Delta y}}{\mathrm{\Delta x}}$$

## Results

Overall, the direction and magnitude of the changes in output variables and curve shapes were within a plausible clinical range in the simulated cases. The specific data for each case is presented below.

### Normal physiology

**Figure 4:**
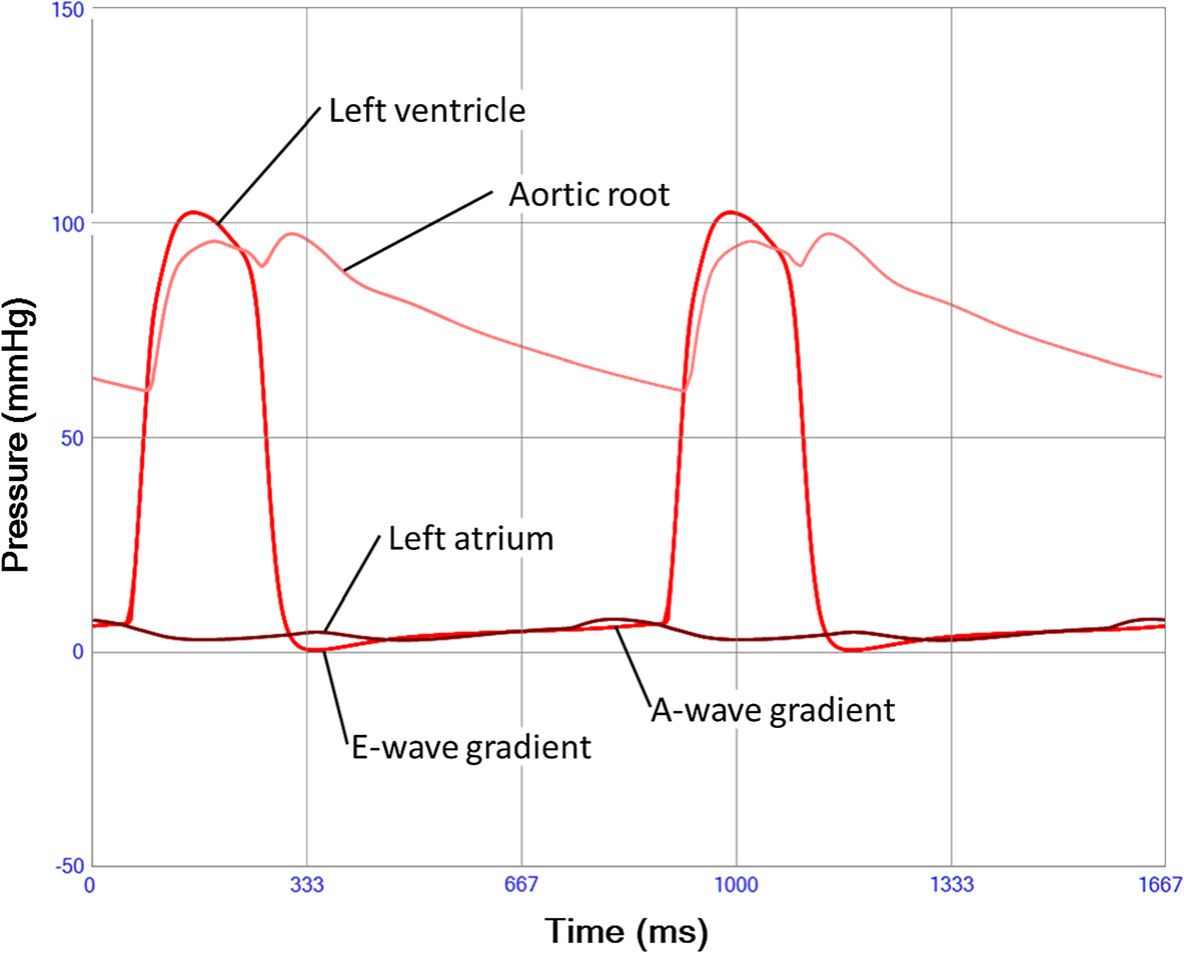
**Left ventricular (red), aortic root (pink) and left atrial pressure (brown) during two heart cycles.** An early diastolic as well as a late positive ventriculo-atrial pressure gradient results in the mitral E and A-waves respectively. A post-ejection aortic pressure incisura is seen corresponding to aortic valve closure followed by a “reflected” wave.

**Figure 5:**
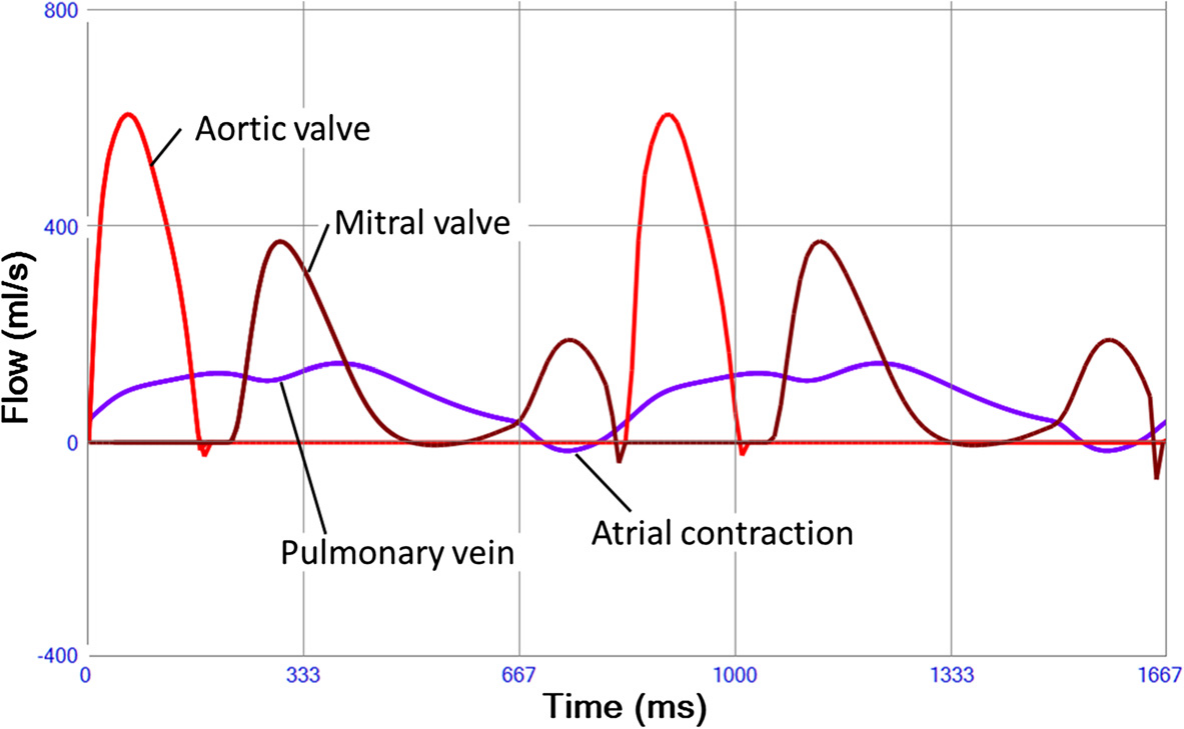
**Aortic valve flow (red), mitral valve flow (brown) and pulmonary vein flow (purple).** Small regurgitant flows are seen when the valves are closing. A minimal reversal of pulmonary vein flow is seen corresponding to the left atrial contraction (mitral A-wave).

Running the model with basal settings representative of a 30 years old healthy man resulted in cardiac output 5.09 l/min, systemic arterial blood pressure 112/61(79) mmHg, pulmonary arterial pressure 24/8(11) mmHg, mean right atrial pressure 4 mmHg and mean left atrial pressure 5 mmHg. The model output of pressures (Figure 4 and Additional file 11: Figure S9), volume-flow rates (Figure 5 and Additional file 12: Figure S10) and timing within the cardiac chambers and the systemic and pulmonary circulatory parts created by the model resembled in pattern and magnitude the ones found in clinical practise (Table 1).

**Table 1:** **Results from a simulation of a healthy man (age 30 years, weight 70 kg, 170 cm) compared to published normal values**[13,25]

**Global**	**Unit**	**Normal values**	**Simulated normal case**
Heart rate	min^-1^	69 ± 17	72
Cardiac output	l · min^-1^	6.3 ± 2.4	5.09
Stroke volume	ml	89 ± 30	71.6
Systolic arterial pressure	mmHg	112/74 (87)	112/61 (79)
Pulmonary arterial pressure	mmHg	22/9 (14)	24/8 (11)
Myocardial volume	ml	374 ± 110	380
Maximum total heart volume	ml	762 ± 133	737
Total blood volume	ml	5105 ± 578	5600
Systemic arterial volume	%	11.4	7.0
Systemic capillary volume	%	5.4	4.4
Systemic venous volume	%	70.0	71.9
Pulmonary blood volume	%	8.1	10.7
Pulmonary capillary volume	%	2.5	2.2
Systemic arterial O_2_ saturation	%	96.4 ± 0.5	96.2
Mixed venous O_2_ saturation	%	70 ± 5	68.3
Coronary sinus O_2_ saturation	%	32 ± 8	28.6
**Left ventricle**			
Ejection fraction	%	59 ± 4	67
Stroke work	mmHg·ml	5600 ± 1000	6735
End-systolic elastance	mmHg·ml^-1^	3.51 ± 1.26	2.43
Preload recruitable stroke work	mmHg	123 ± 36	88.8
dp/dt_max_	mmHg·s^-1^	1840 ± 327	2359
dp/dt_min_	mmHg·s^-1^	−1864 ± 390	−2666
Tau	ms	33 ± 8	18
End-diastolic volume	ml	123 ± 28	106.8
LA volume min --- max	ml	29.9 --- 76.7	55 --- 81
LAP	mmHg	8.3 ± 2.5	4.8
LV Tei	---	0.39 ± 0.05	0.41
LV Ees/Ea	---	1.62 ± 0.80	1.78
**Right ventricle**			
RV EF	%	61.0 ± 5.8	68
RVSW	mmHg·ml	1980	1706
RV E_es_	mmHg·ml^-1^	0.66	0.51
RV dpdt_max_	mmHg·s^-1^	422 ± 46	538
RV dpdt_min_	mmHg·s^-1^	−355 ± 45	−289
RV Tau	ms	---	33
RV EDV	ml	111 ± 22	105.6
RA volume min --- max	ml	---	49 --- 78
RAP	mmHg	4.8 ± 2.4	3.7
RV Tei	---	0.28 ± 0.05	0.13
RV Ees/Ea	---	---	1.14

Values are presented as mean ± standard deviation when available.

The shapes and areas (stroke work) of the ventricular pressure-volume loops (Additional file 13: Figure S11a) as well as right and left ventricular ejection fraction were within normal limits [25] (Table 1). The atrial loops (Additional file 13: Figure S11b) were similar to the scarce published data [26].

### Systolic heart failure

**Figure 6:**
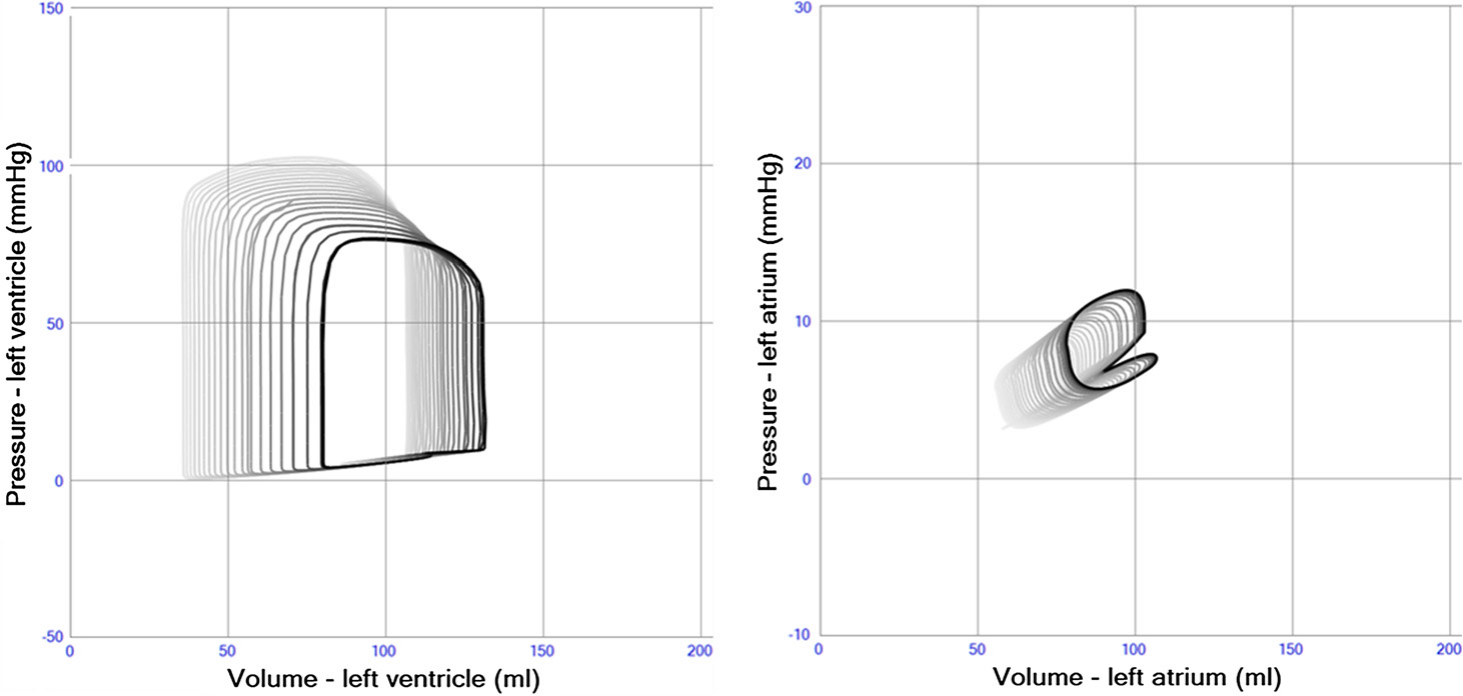
**Intracardiac pressure-volume loops from the left ventricle (left panel) and left atrium (right panel) in simulation of pure systolic left heart failure (E**_**max **_**2.8 → 1.0).** Stroke volume and blood pressure decrease. Left ventricular and atrial volumes as well as filling pressures increase.

The simulation of systolic heart failure resulted in decreased left ventricular ejection fraction, stroke work, stroke volume and systolic pressure, while left ventricular end-diastolic volume and filling pressures increased (Figure 6 and Table 2).

**Table 2:** Key observations during simulation of pathological states

**Case**	**HR**	**SAP**	**LAP**	**LVEF**	**LVSV**	**LVSW**	**LVEDV**	**LVESP**
	min^-1^	mmHg	mmHg	%	ml	mmHg·ml	ml	mmHg
Normal physiology	72	112/61(79)	4.8	67	71	6735	107	94
Systolic heart failure	72	84/51(62)	8.1	39	52	3499	133	77
Diastolic heart failure	72	90/53(66)	9.2	69	66	4194	83	72
Aortic stenosis	72	89/55(68)	7.1	46	58	9117	126	181
Aortic regurgitation	72	110/42(65)	7.1	76	101	8842	133	83

*HR* heart rate, *SAP* systemic arterial pressure, *LAP* left atrial pressure, *LVSV* left ventricular stroke volume, *LVSW* left ventricular stroke work, *LVEDV* left ventricular end diastolic volume, *LVESP* left ventricular end systolic pressure.

### Diastolic heart failure

**Figure 7:**
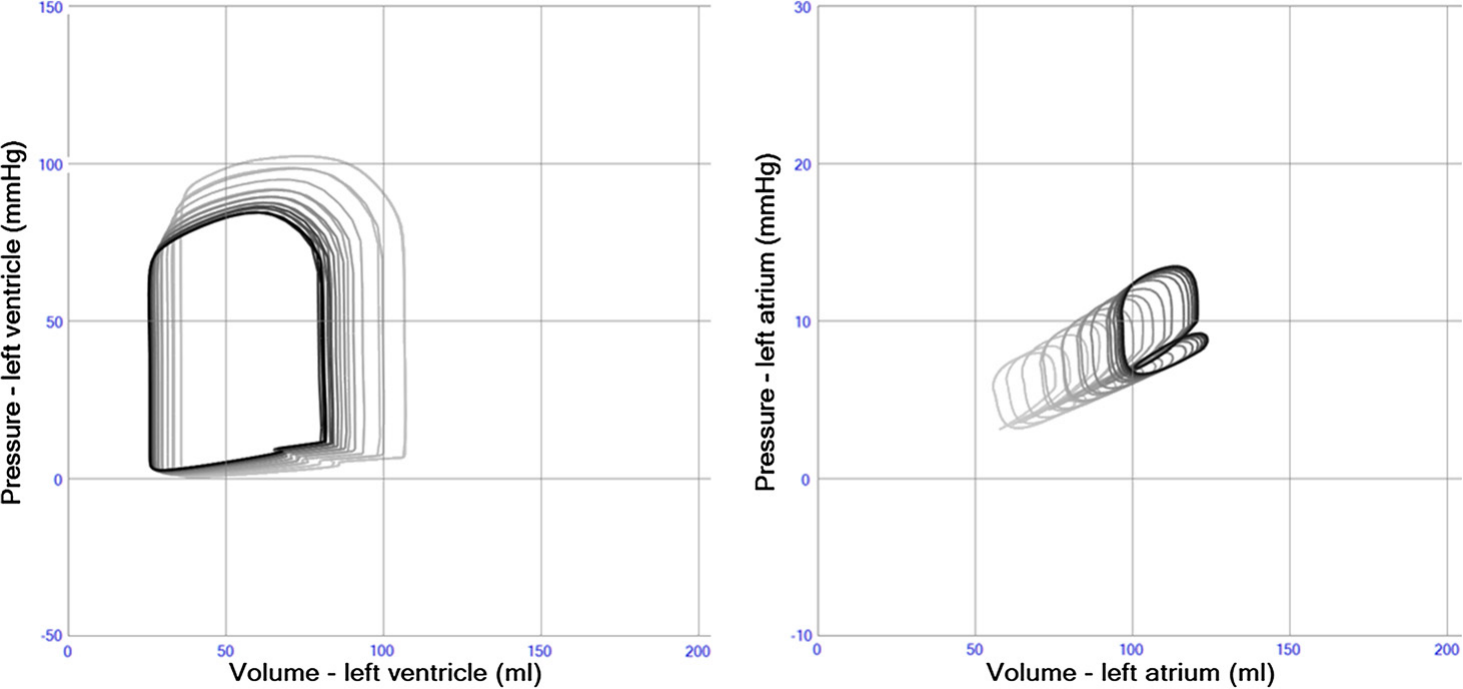
**Intracardiac pressure-volume loops from the left ventricle (left panel) and left atrium (right panel) in simulation of diastolic heart failure (E**_**min **_**0.05 → 0.12).** Stroke volume and blood pressure decrease. Left ventricular volume decreases, while left atrial volume and filling pressures increase.

The simulation of diastolic heart failure showed preserved left ventricular ejection fraction, but decreased stroke work, end-diastolic volume, stroke volume and systolic pressure, while left ventricular filling pressures increased (Figure 7 and Table 2).

### Aortic stenosis

**Figure 8:**
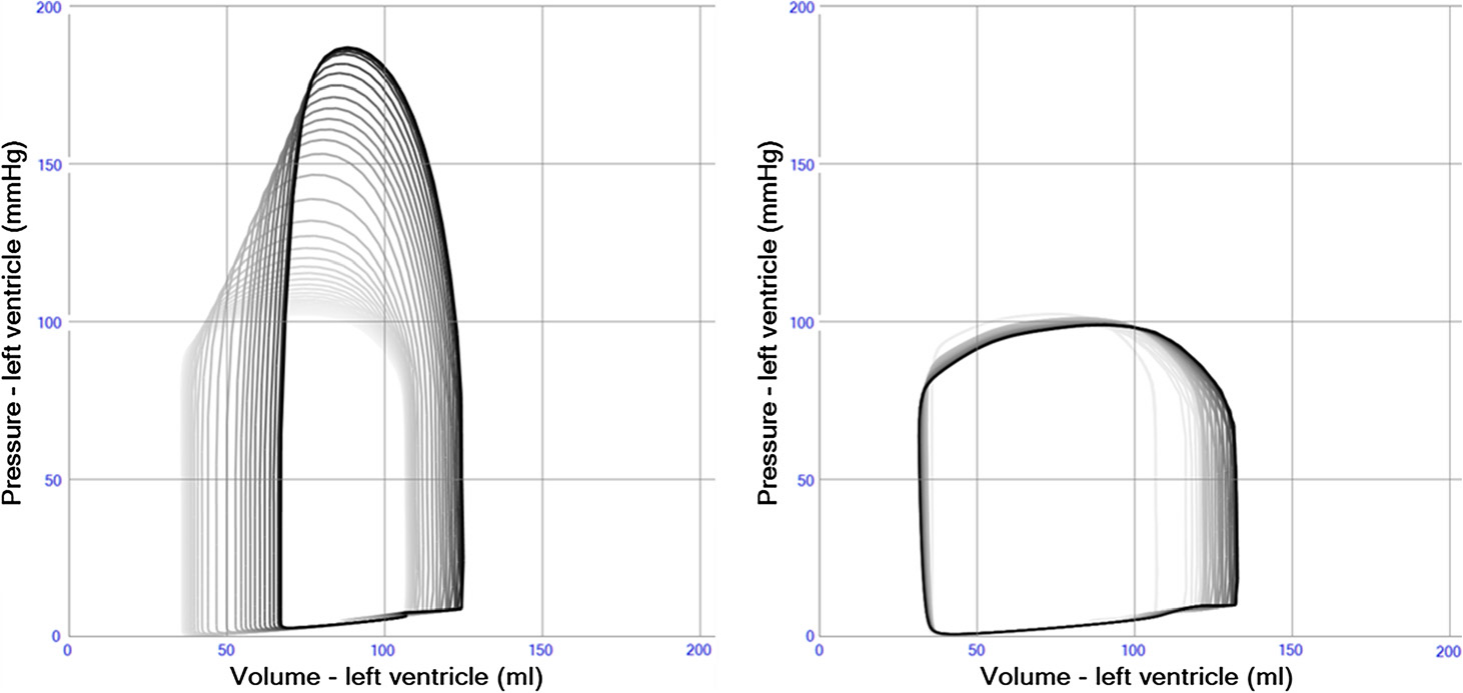
**Intracardiac pressure-volume loops from the left ventricle in simulation of aortic stenosis (open area 5.0 → 0.7 cm**^**2**^**, left panel) and aortic regurgitation (closed area 0.0 → 0.2 cm**^**2**^**, right panel.** Stroke volume decreases in aortic stenosis despite high intraventricular pressure. Apparent stroke volume increases and systemic blood pressure decreases in aortic regurgitation. Left ventricular end-diastolic pressure and volume increase in both scenarios.

The simulation of aortic stenosis resulted in decreased left ventricular ejection fraction, stroke volume and systemic blood pressure, although left intraventricular pressure and stroke work increased (pressure-load). Left ventricular end-diastolic volume and filling pressures increased (Figure 8 and Table 2).

### Aortic regurgitation

Simulated data of aortic regurgitation showed apparent left ventricular ejection fraction and stroke volume increase, systemic blood pressure decrease although stroke work increased (volume-load). Left ventricular end-diastolic volume and filling pressures increased (Figure 8 and Table 2).

### Valsalva

**Figure 9:**
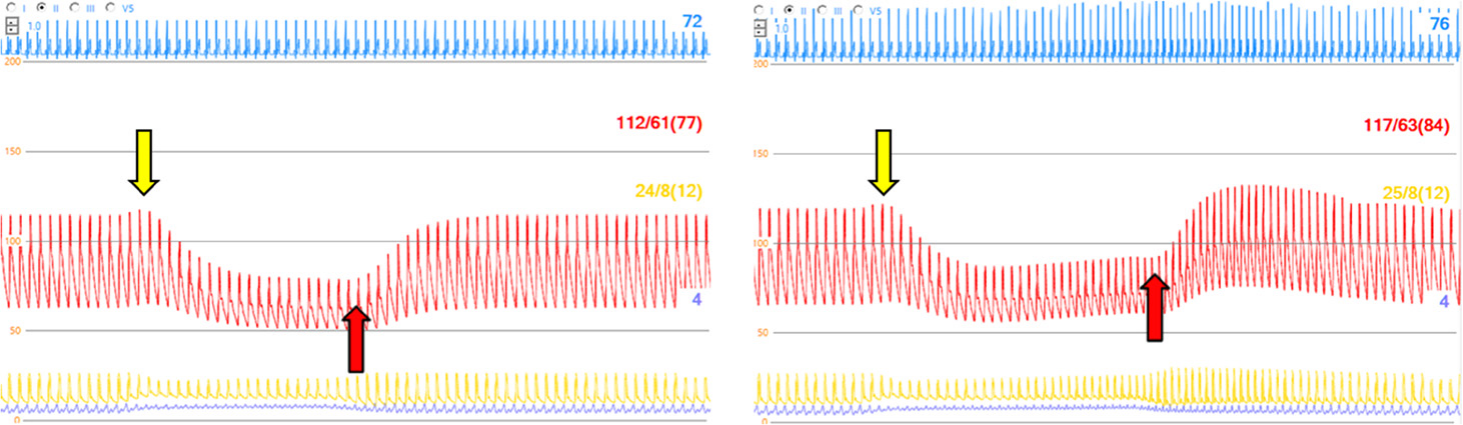
**Simulation of Valsalva maneuver (increase in intrathoracic pressure from zero to +10 mmHg between arrows) without (left panel) and with (right panel) baroreceptor reflex activated.** ECG (blue), arterial pressure (red), pulmonary arterial pressure (orange) and central venous pressure (blue), pressure increase when increasing intrathoracic pressure (yellow arrow), decrease when releasing pressure (red arrow). The baroreceptor reflex increase heart rate, left ventricular contractility and systemic arterial resistance. Blood pressure is better maintained and a pressure overshoot is seen after release of the pressure.

The simulation of a Valsalva maneuver without and with activated baroreceptor reflex is shown in Figure 9. The sudden decrease in afterload with preserved preload when increasing intrathoracic pressure [27] is the explanation for the initial rise in arterial pressure as indicated in the figure. The opposite effect was seen immediately after decreasing intrathoracic pressure. Arterial blood pressure was as expected better preserved when the baroreceptor reflex was activated due to increase in heart rate, ventricular contractility and systemic arterial resistance.

### Exercise

**Figure 10:**
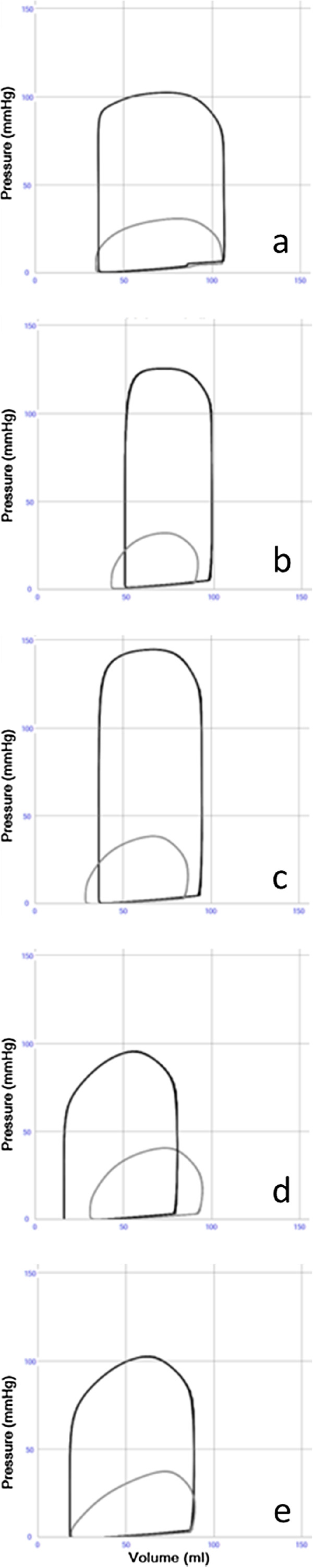
**a-e. Pressure volume loops in left (black) and right (gray) ventricle during stepwise simulation of moderate exercise. (a)**. Normal resting state. HR 72/min. CO 5.11 l/mint.SAP112/61(79). PAP 24/8(12). **(b)**. Increase in heart rate +100%.HR 144/min. CO 7.08 l/mint.SAP132/86(102). PAP 26/9(14). **(c)**. Increase in right and left ventricular systolic function +50%. HR 144/min. CO 8.41 l/mint SAP153/95(117). PAP 29/9(15). **(d)**. Decrease in systemic arterial resistance. Increase in systemic arterioli diameter +50%. HR 144/min. CO 9.23 l/mint.SAP 86/48(60). PAP 30/9(15). **(e)**. Decrease in pulmonary arterial resistance.Increase in pulmonic arterioli diameter +50%. HR 144/min. CO 10.18 l/mint.SAP 94/50(65). PAP 27/5(13).

The stepwise simulation of exercise is shown in Figure 10. Starting from a resting state, heart rate and cardiac contractility is increased followed by reductions in systemic and pulmonary vascular resistance resulting in a total cardiac output above 10 l/min.

### Arteriosclerosis

**Figure 11:**
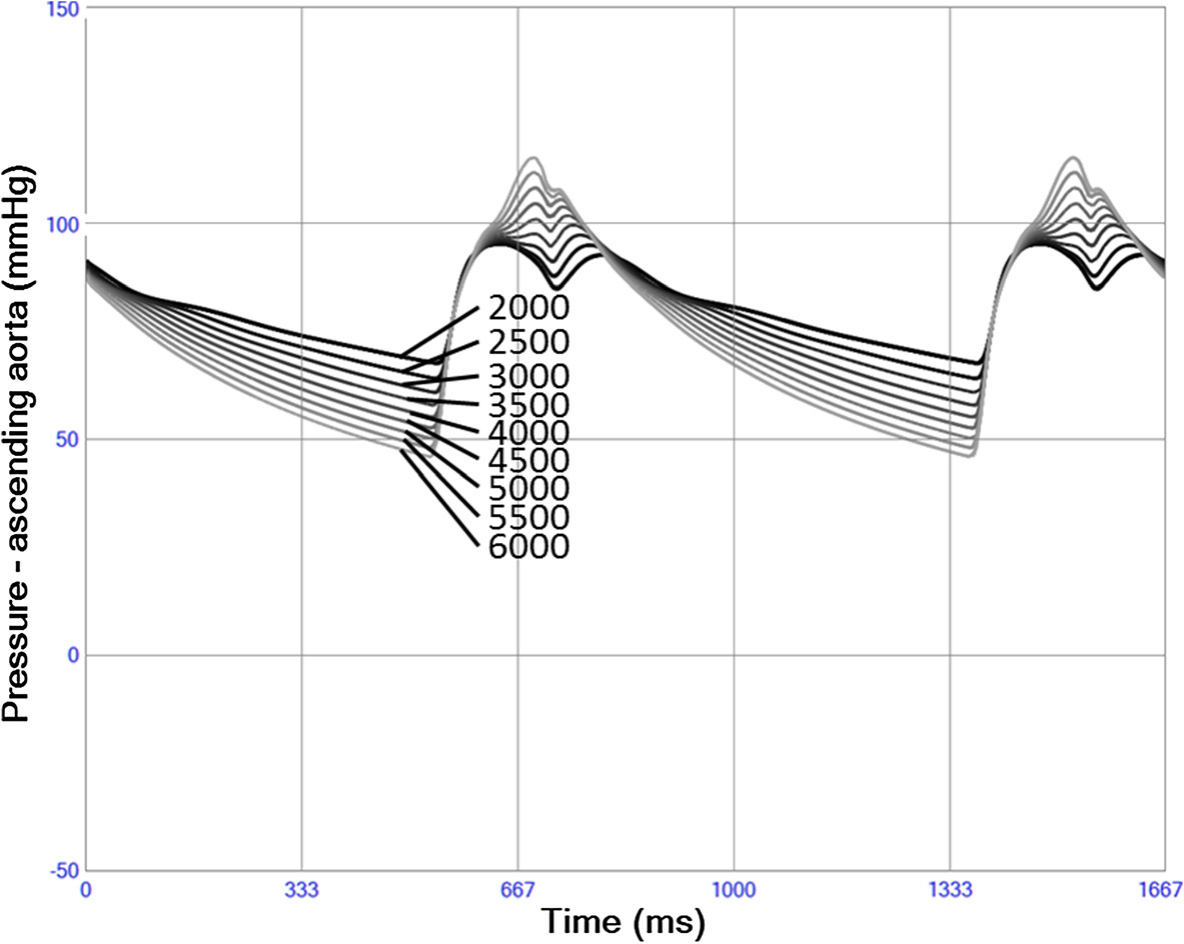
**Blood pressure in ascending aorta with changing vascular stiffness.** When increasing (lighter) Young’s elastic modulus Y systolic pressure increases and diastolic pressure decreases. The increased stiffness also results in a shorter systolic ejection due to a premature arterial recoil/reflection. The increase in afterload also results in decreasing left ventricular stroke volume despite increasing stroke work.

The presence of arteriosclerosis, simulated by increased vascular stiffness, resulted in increased systolic aortic pressure and decreased diastolic aortic pressure. A decrease in systolic ejection time during this process is indicated by the premature arrival of the reflected pressure wave changing the shape of the pressure curves in a characteristic way (Figure 11).

### Sensitivity analysis

Blood volume was the single most important parameter in the sensitivity analysis (Additional file 10). The pericardial volume constant v_pc0 was second most important. Pulmonary arteriolae radius (radius_pur) was more important than systemic arteriolae radius (radius_r). Right ventricular passive diastolic stiffness (e0_rv) and systolic contractility (emax_rv) was more important than their left sided equivalents (e0_lv and emax_lv, respectively). Parameters with less than 10% sensitivity are not shown.

## Discussion

This work presents a closed-loop real-time lumped parameter simulation model of the cardiovascular system where normal physiology and oxygen transport as well as systolic heart failure, diastolic heart failure, valve failure, intracardiac shunts and pulmonary failure can be simulated separately or in combination.

The validation and appreciation of clinical relevance of the model is based on empirically relevant changes in simulated clinical parameters when being compared with measurements made by clinically available methods such as intravascular pressure catheters and ultrasound. It is not possible to validate the large number of parameters unambiguously with available clinical and/or experimental data, but when taking the complexity of the system into account and the variety of disease states that can be simulated, the clinical relevance becomes a powerful validation and hypothesis creating tool. Another approach to challenge the adequacy of a cardiovascular simulation model is to study if model responses are adequate when evaluating model output in new scenarios with pathological states not previously tested during construction and tuning of the model. We have chosen to present model output illuminating a range of normal and pathophysiological states, illustrating the wide scope of the model. The normal resting state, the Valsalva maneuver and physiological changes during exercise are parts of normal physiology that should be handled correctly by a multi-purpose cardiovascular simulation model. The pathophysiological states chosen represent important clinical problems with various backgrounds. Notable is that very few parameter changes are needed to simulate the basic features of these states. Further parameter changes are needed to simulate secondary compensatory adaptations such as e.g. ventricular hypertrophy in aortic stenosis. This can be automatized in a simulation model [5], but manual stepwise adaptation often enables a better understanding of the relation between the primary problem and its consequences. The lack of secondary changes such as blood volume increase and systemic vasoconstriction usually seen in clinical cases with low cardiac output can also explain the small increase in filling pressures seen in simulation of heart failure and aortic valve failure in the present work.

The combined effect of elastic recoil in large elastic arteries and reflection of pressure waves in the peripheral vasculature is of importance in coronary physiology as well as in determining left ventricular afterload. A distributed lumped parameter model as presented in this article is usually not considered adequate for describing wave transmission and reflection in the vascular tree, although the use of an inductor/inertia in each vascular compartment and a high resistance in peripheral components create pressure and flow curves with realistic amplitudes and time delays resembling effects of reflected waves (Figures 4, 5 and 11). The advantage with our approach is less demanding calculations enabling real-time simulation and also the possibility to connect artificial circulatory support to any node in the model as will be explored in future works.

Aortic characteristic impedance is originally defined as a single purely resistive component describing the properties of the entire vascular tree in the high frequency domain, when seen from the aortic valve [20]. Placing this resistor in line with the blood flow, as is usually done in the 3-compartment Windkessel model, gives adequate results at high frequencies, but obscures the meaning of the more basic concept "total peripheral resistance" in the low frequency domain [19]. When placing the resistor in line with the capacitor in parallel to the blood flow in each vascular compartment as is done in the present model (Additional file 14: Figure S2), this component still fulfills the role of the characteristic impedance in the high-frequency domain without affecting the total peripheral resistance. This is the reason why the characteristic impedance is calculated according to Equation 8. Furthermore it has a physical meaning representing the damping effect imposed by the viscous properties of the vascular wall (non-linear Maxwell viscoelastance model), although the detailed relation to tissue properties remains to be elucidated.

The algorithms for scaling of the cardiac elastance functions with changing heart rate seem efficient up to a frequency of 160 min^-1^. Above this heart rate cardiac output decreases suggesting either suboptimal diastolic heart function (in the model) or suboptimal scaling of the elastance functions. It is also possible that atrioventricular (AV)-plane motion, not included in the present model, is necessary to improve ventricular filling during tachycardia in real life physiology.

Sensitivity analysis in a non-linear model should ideally be performed for every possible set of input parameters. With many input parameters it is neither feasible to perform the calculations nor to interpret them in every possible model state. The choice of relevant parameters therefore must rely on physical and physiological model relations; however the relevance of the model is supported by the performed sensitivity analysis in normal physiology, where parameters determining cardiac filling, afterload and ventricular systolic performance are most important for cardiac flow and pressure generation (Additional file 10).

### Limitations and future development of the current model

The scientific, clinical and educational value of the model is the possibility to study combinations of physiological and pathophysiological cardiac and vascular properties as well as the effects of potential modifications or treatments. This holistic approach enables hypothesis generation regarding the direction of changes and qualitative relations between parameters in a wide variety of normal and pathological states suggesting possible usefulness as a clinical decision support tool. Detailed quantitative conclusions are however not justified without further validating studies. It must also be kept in mind that differences in the magnitude of changes between model output and patients will persist due to model simplifications and non-modelled parameters. Patient related predictions or hypotheses created by any simulation model have to be weighed against other sources of knowledge such as clinical studies and experience.

The mathematical functions creating the time-varying elastance functions are not based directly on cellular or mechanical properties of the myocardium, but should rather be seen as a convenient and well-established way to mimic a realistic physiological ventricular filling and contraction pattern during variable loading conditions in a computer model. The chosen functions are scalable with changes in cardiac mechanical properties and heart rate.

Future improvements of the cardiac model aim at adjustment to known physiological relations, including the AV-plane movement contributions to the cardiac pumping mechanism [28] and scaling of the model to neonatal and pediatric size. A specific aim is also to develop and validate simulated hemodynamic responses in patients treated with extra-corporeal circulation during intensive care, such as left ventricular unloading during support with mechanical left ventricular assist devices and systemic oxygen delivery and pulmonary hypertension in congenital cardiopulmonary anomalies. Further development of the model will also aim at implementing a semi-automatic adaptation to available patient-specific clinical data and from this enable prediction of the hemodynamic results from possible surgical and pharmacological interventions.

During extreme conditions such as cardiac arrest, extreme hypovolemia or volume overload, when pressures are outside a normal range an improved model including simulation of vascular collapse and overdistension needs to be developed. Moreover, due to the lack of true 3D properties some pathology, *e.g.* myocardial ischemia and infarctions with regional cardiac dysfunction, cannot be represented in detail. The same problem is inherent in many cardiac arrhythmias involving aberrant activation of the myocardium.

## Conclusions

The results of the present publication show that it is possible to build a real-time computer simulation model illustrating the cardiovascular system, including oxygen transport, as evidenced by the base-line values almost entirely within reference limits of normal physiology and by relevant direction of changes in clinical parameters in a variety of pathophysiological states. The understanding of complex hemodynamic states may be further improved when known model parameter changes are studied and simultaneously compared in detail by the user with clinical data.

## Abbreviations

L: Blood flow inertance; ρ: Density; l: Valve inflow length; A: Area; Esv: Ventricular septal stiffness; Esv0: Ventricular septal stiffness constant; elv: Left ventricular systolic elastance; plv: Left ventricular pressure; vlv: Left ventricular volume; Prv: Right ventricular pressure; Q: Flow; ∆P: Pressure gradient; C: Gorlin empirical constant; R0: Resistance at normal mean pressure; I0: Inertia at normal mean pressure; E0: Elastance at normal mean pressure; P0: Normal mean pressure; n: Number of parallel vessels in vascular compartment; η: Blood viscosity; l: Vessel length; r0: Vessel radius at normal mean pressure; h: Vessel thickness; Yinc: Young’s modulus; Ω: Vascular wall resistance; λ: Vascular wall resistance scaling factor; E: Elastance; Hb: Hemoglobin; Sat: Oxygen saturation; Volume: Model compartment volume; Emin: Basal cardiac chamber passive stiffness; LVESP: Left ventricular end-systolic pressure; RVESP: Right ventricular end-systolic pressure; LAP: Left atrial pressure; RAP: Right atrial pressure; LVSW: Left ventricular stroke work; RVSW: Right ventricular stroke work; CO: Cardiac output; S: Sensitivity; y: Output variable; x: Input parameter; BloodVolume: Blood volume; v_pc0: Pericardial volume constant; Radius_pur: Pulmonary arteriolae radius; Radius_r: Systemic arteriolae radius; e0_rv: Right ventricular passive diastolic stiffness; emax_rv: Right ventricular systolic contractility; HeartRate: Heart rate; Alpha2_rv: Duration of right ventricular relaxation; Radius_a: Systemic arterial radius; e0_lv: Left ventricular passive diastolic stiffness; MeanPressure_v: Normal mean pressure in small systemic veins; Radius_puc: Pulmonary capillary radius; emax_lv: Left ventricular systolic contractility; Radius_rcardx: Right carotid arterioli radius; Radius_rcarsin: Left carotid arterioli radius; Radius_pusv: Pulmonary small vein radius; Number_pur: Pulmonary arterioli number; Length_pur: Pulmonary arterioli length; Length_r: Systemic arterioli length; lambda_rv: Right ventricular stiffness constant; Number_r: Systemic arterioli number; AV: Atrio-ventricular; dp/dtmax: Maximum pressure derivative; dp/dtmin: Minimum pressure derivative; Tau: Ventricular relaxation constant Tau; EDV: Left ventricular end-diastolic volume; LA volume: Left atrial volume; Tei: Tei index = Myocardial performance index; Ees/Ea: Ventricular-vascular coupling; RA volume min --- max: Right atrial volume; HR: Heart rate; SAP: Systolic arterial pressure; LVEF: Left ventricular ejection fraction; LVSV: Left ventricular stroke volume; LVEDV: Left ventricular end-diastolic volume; ECG: Electrocardiogram; PAP: Pulmonary arterial pressure

## Competing interests

Michael Broomé is the founder and owner of the company Aplysia Medical AB developing the simulation software Aplysia CorVascSim. There are no other conflicts of interest.

## Authors’ contributions

MB constructed the model, performed programming, sensitivity analysis and drafted the manuscript. EM and AB participated in model construction and adaptation of the model to engineering standards as well as in manuscript drafting. BJS participated in model development in relation to previously published models and drafting of the manuscript. MB, BF and BJS all participated in evaluation of the clinical relevance of the model as being clinically active medical doctors. All authors read and approved the final manuscript.

## Supplementary Material

Additional file 1: Figure S1. Electrical analogue sketch showing both the heart and the vascular system. The dark yellow area is the pericardium containing the cardiac chambers and coronary vessels. The light yellow area is the intrathoracic space containing the pericardium, the pulmonary circulation and the thoracic aorta. Click here for file

Additional file 2 **Closed-loop real-time simulation model of hemodynamics and oxygen transport in the cardiovascular system.** Supplement. Click here for file

Additional file 3: Figure S3 Left (black thick) and right (gray thick) ventricular time-varying elastance functions during two heart cycles. Atrial elastance functions are shown with thin lines. The amplitude is closely related to contractile function while the volume-dependent basal level describes passive stiffness. Click here for file

Additional file 4: Figure S4 The ECG and valvular areas simulated during two normal heart beats. ECG (light blue) and area-changes during the heart cycle for aortic (red), pulmonary (yellow), mitral (brown), tricuspid (blue) valves are shown. Click here for file

Additional file 5: Figure S5 Modeled volume changes (black) due to pressure dependent ventricular septal volume shift from left to the right ventricle during two heart cycles. Septal stiffness values of 40, 30, 20 and 10 mmHg/ml are shown. Lower septal stiffness increases septal shift. ECG (blue) is shown as reference. Click here for file

Additional file 6: Figure S6 Simulated pressure gradients over the mitral valve during diastole. The total mitral pressure gradient (thick black) is decomposed into a dominating Bernoulli gradient (grey) and an inertial component (dotted black). Click here for file

Additional file 7: Figure S7 Pericardial pressure-volume relations. The black curve illustrates the relation used in the present model with the range used by a single heart beat in orange. Pericardial pressure is slightly negative. Click here for file

Additional file 8: Figure S12 Pressure-volume relations in arterial vascular segments illustrating non-linear stiffness in the actual working pressure range of each vascular segment in accordance with Equation 10. Click here for file

Additional file 9: Figure S8 Blood flow in left (black) and right (gray) coronary artery during two heart cycles. Left coronary artery blood flow decreases during systole due to vascular compression caused by high left ventricular wall stress. Click here for file

Additional file 10 Sensitivity analysis. Click here for file

Additional file 11: Figure S9 Pressure changes simulated during two heart cycles in the aortic root (black), ascending aorta, proximal and distal aortic arch, descending aorta and a peripheral artery (light gray). The segments between the aortic root and the peripheral artery are shown in different shades of gray. Click here for file

Additional file 12: Figure S10 Flow pulsatility in pulmonary capillaries (pink) is larger than in the systemic capillaries (green). Pulmonary valve flow (orange) and tricuspid valve flow (blue) resembling left-sided flows are also shown. Click here for file

Additional file 13: Figure S11 a. Intracardiac pressure-volume loops from the left ventricle (black) and right ventricle (gray). b. Intracardiac pressure-volume loops from the left atrium (black) and right atrium (gray). Click here for file

Additional file 14: Figure S2 Electrical analogue sketches showing details of the heart chambers, valves and vascular compartments. The arrows show directions of blood flow. e(t); time-varying elastance representing systolic and diastolic chamber properties, R_wall_; a resistance representing viscous chamber wall properties, R_outflow_; a linear chamber outflow resistance, B; a non-linear Bernoulli valve resistance, I_valve_; an inductance representing flow inertia,, R; a linear resistance, I; an inductance representing flow inertia, C; a non-linear capacitor representing vascular elasticity, Ω; a non-linear resistance term representing viscous vascular wall properties. Click here for file
