# Supplementary figures and images for: Closed-loop real-time simulation model of hemodynamics and oxygen transport in the cardiovascular system
Source: Biomed Eng Online. 2013 Jul 10;12:69. doi: 10.1186/1475-925X-12-69 (PMC3751725; doi:10.1186/1475-925X-12-69)

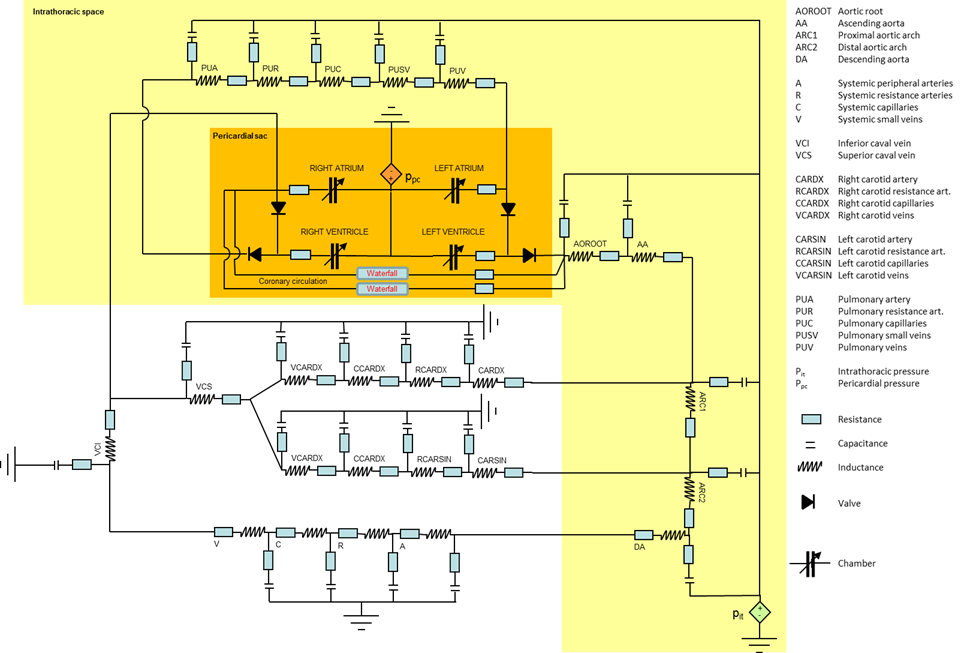

Supplement: Additional file 1: Figure S1. — Electrical analogue sketch showing both the heart and the vascular system. The dark yellow area is the pericardium containing the cardiac chambers and coronary vessels. The light yellow area is the intrathoracic space containing the pericardium, the pulmonary circulation and the thoracic aorta. [file 1475-925X-12-69-S1.tiff]

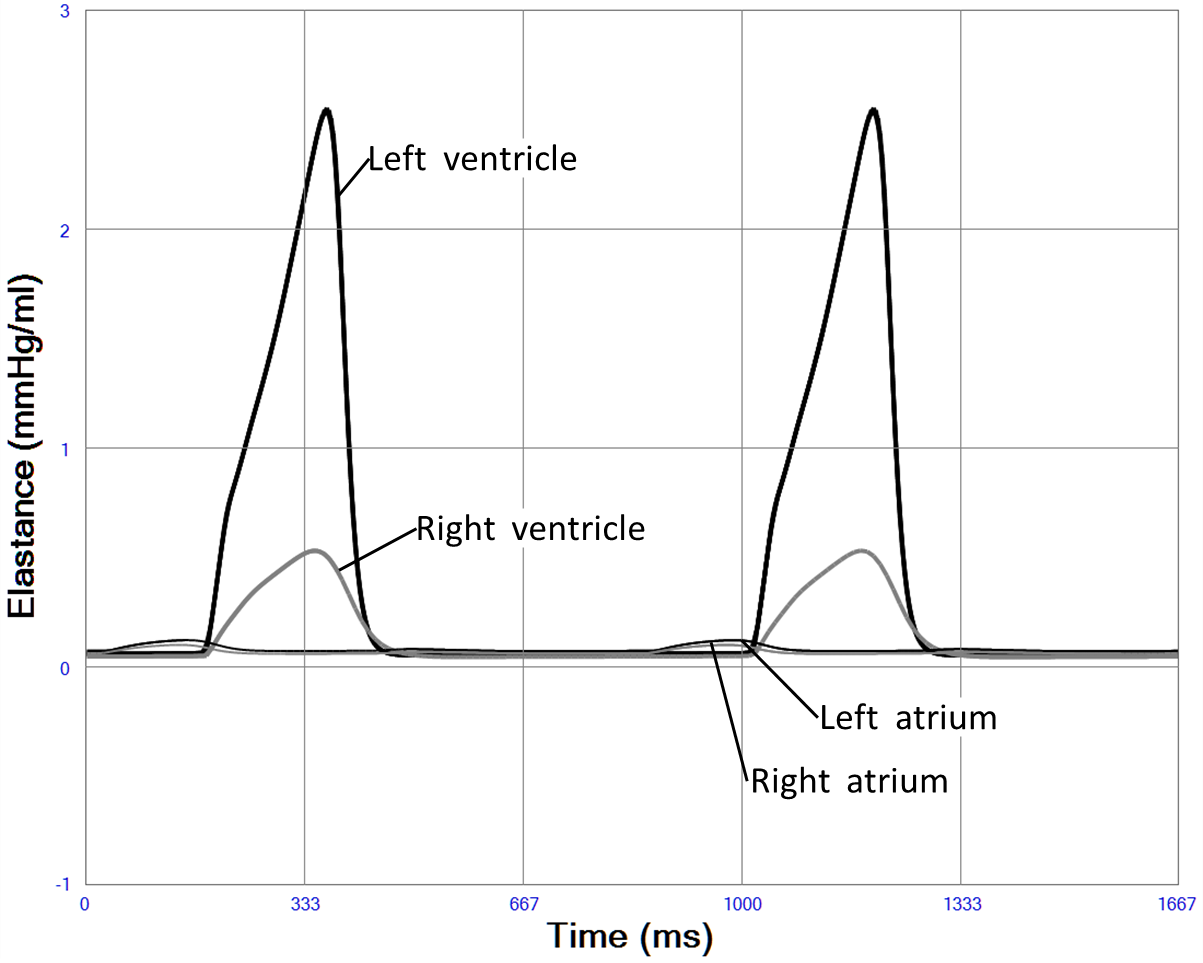

Supplement: Additional file 3: Figure S3 — Left (black thick) and right (gray thick) ventricular time-varying elastance functions during two heart cycles. Atrial elastance functions are shown with thin lines. The amplitude is closely related to contractile function while the volume-dependent basal level describes passive stiffness. [file 1475-925X-12-69-S3.tiff]

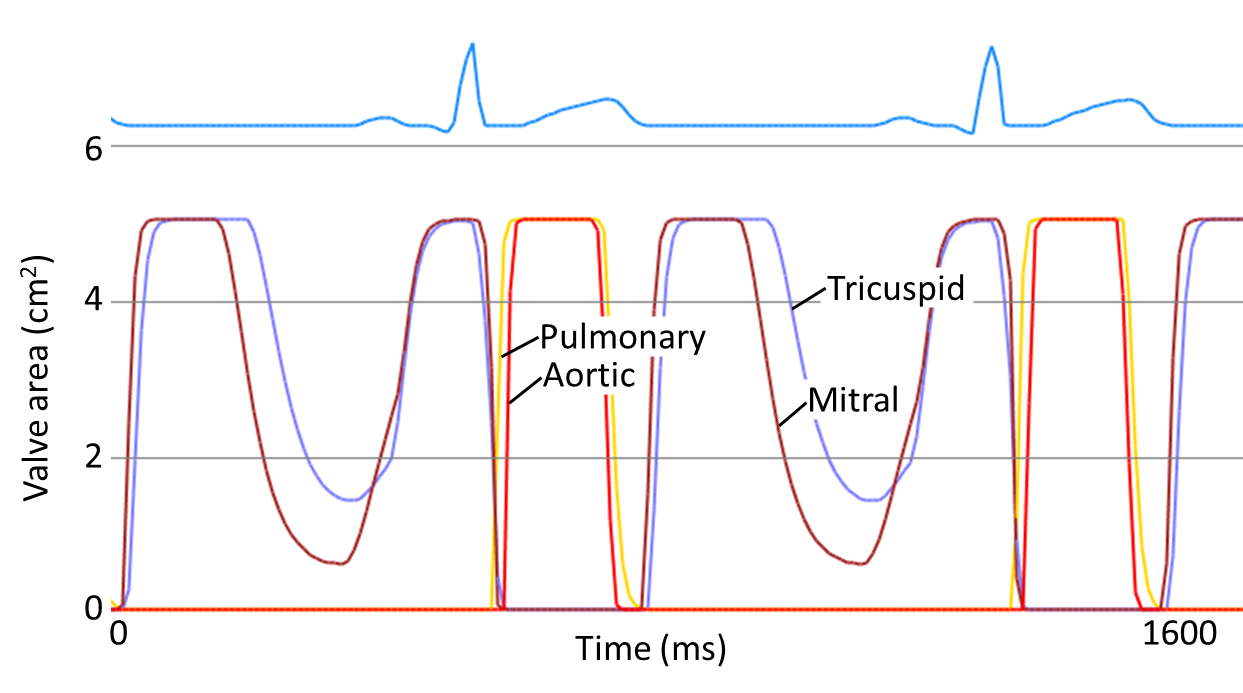

Supplement: Additional file 4: Figure S4 — The ECG and valvular areas simulated during two normal heart beats. ECG (light blue) and area-changes during the heart cycle for aortic (red), pulmonary (yellow), mitral (brown), tricuspid (blue) valves are shown. [file 1475-925X-12-69-S4.tiff]

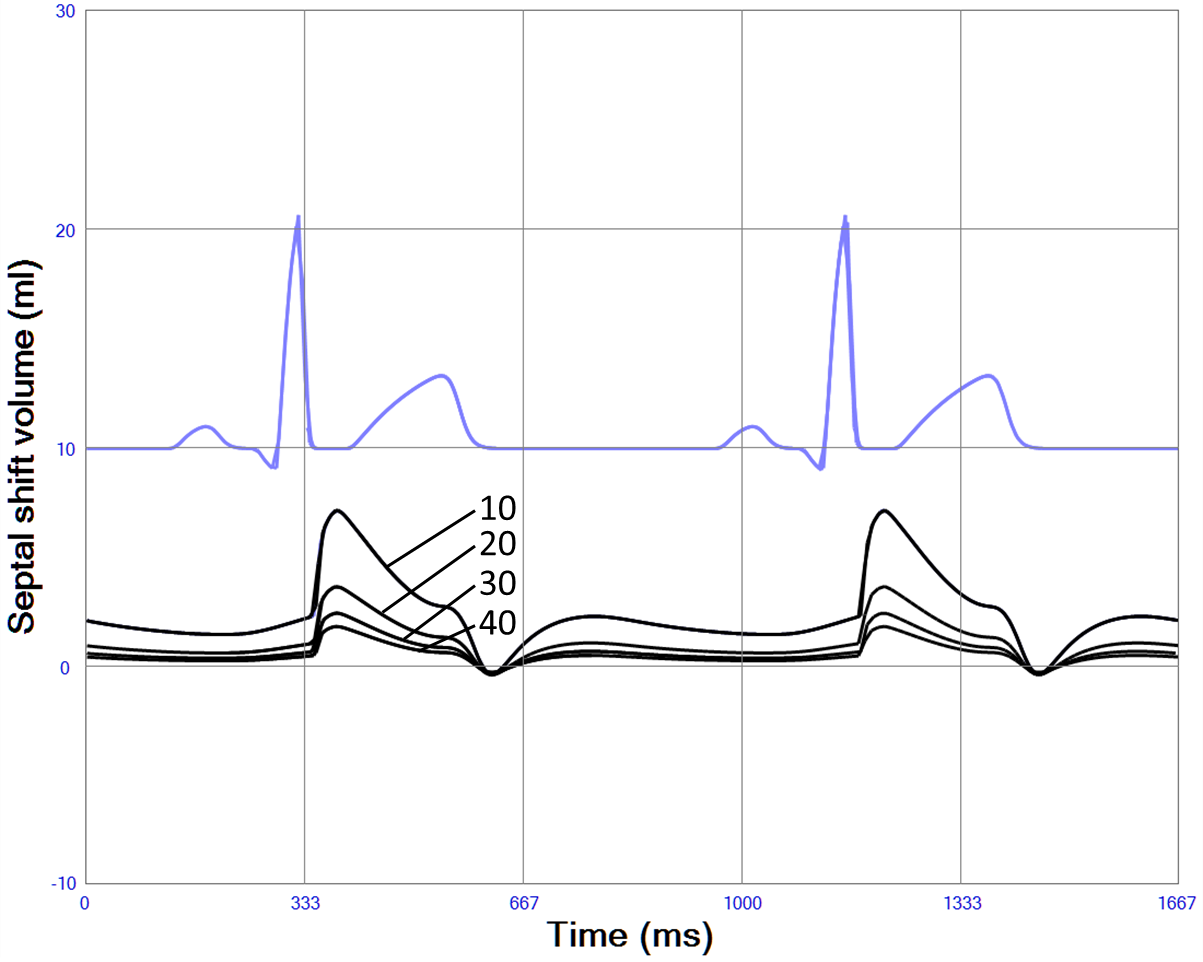

Supplement: Additional file 5: Figure S5 — Modeled volume changes (black) due to pressure dependent ventricular septal volume shift from left to the right ventricle during two heart cycles. Septal stiffness values of 40, 30, 20 and 10 mmHg/ml are shown. Lower septal stiffness increases septal shift. ECG (blue) is shown as reference. [file 1475-925X-12-69-S5.tiff]

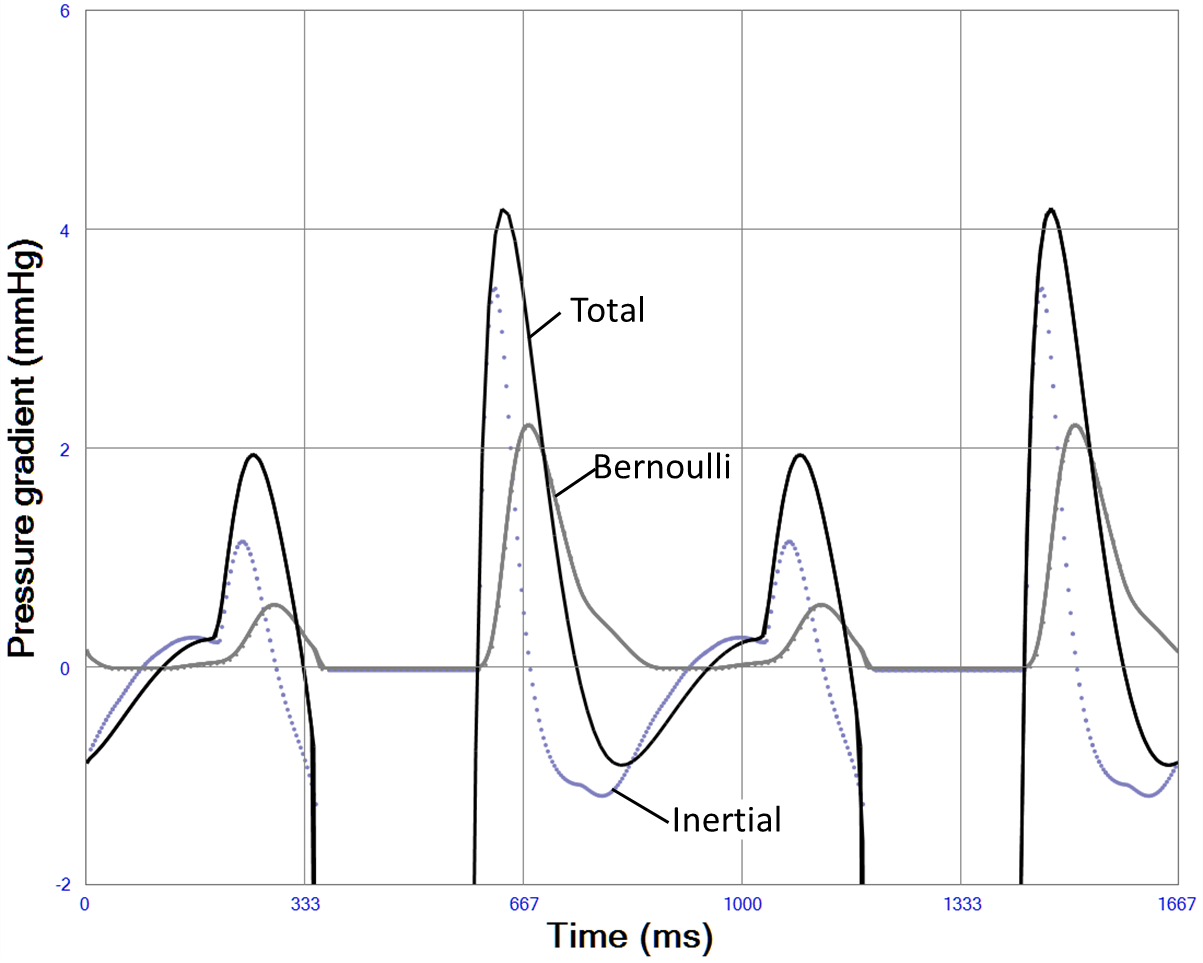

Supplement: Additional file 6: Figure S6 — Simulated pressure gradients over the mitral valve during diastole. The total mitral pressure gradient (thick black) is decomposed into a dominating Bernoulli gradient (grey) and an inertial component (dotted black). [file 1475-925X-12-69-S6.tiff]

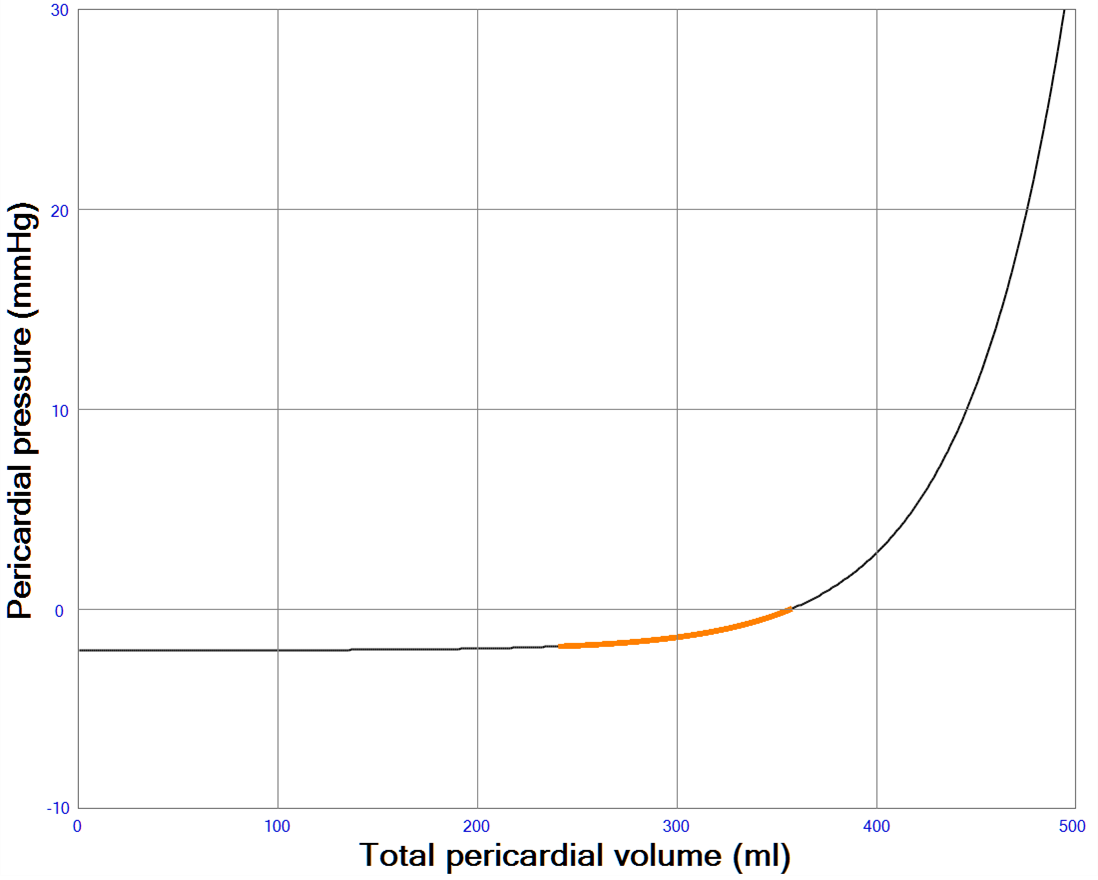

Supplement: Additional file 7: Figure S7 — Pericardial pressure-volume relations. The black curve illustrates the relation used in the present model with the range used by a single heart beat in orange. Pericardial pressure is slightly negative. [file 1475-925X-12-69-S7.tiff]

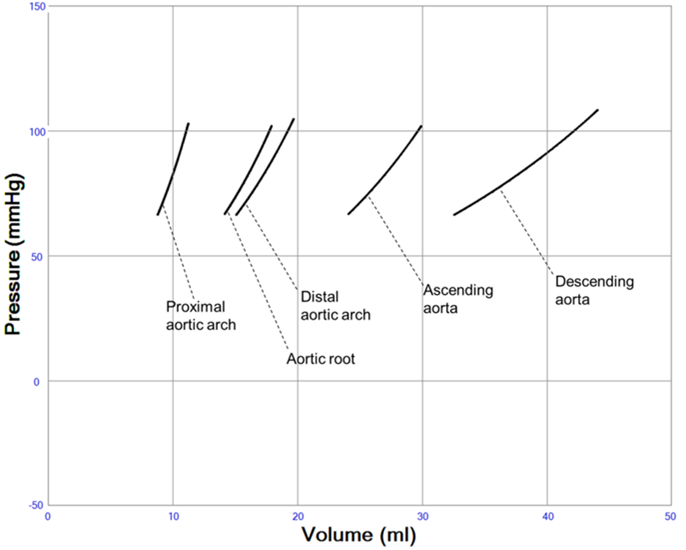

Supplement: Additional file 8: Figure S12 — Pressure-volume relations in arterial vascular segments illustrating non-linear stiffness in the actual working pressure range of each vascular segment in accordance with Equation 10. [file 1475-925X-12-69-S8.tiff]

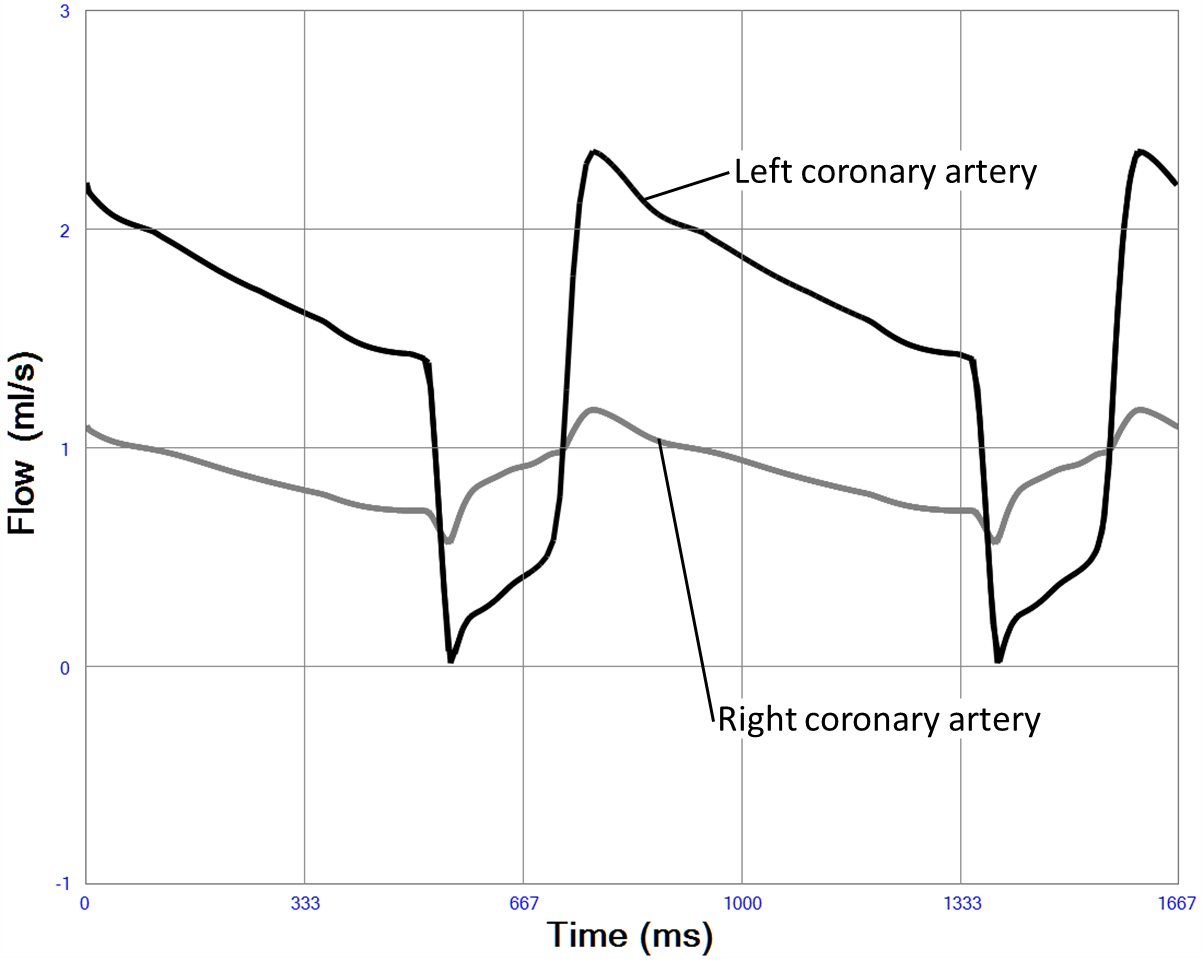

Supplement: Additional file 9: Figure S8 — Blood flow in left (black) and right (gray) coronary artery during two heart cycles. Left coronary artery blood flow decreases during systole due to vascular compression caused by high left ventricular wall stress. [file 1475-925X-12-69-S9.tiff]

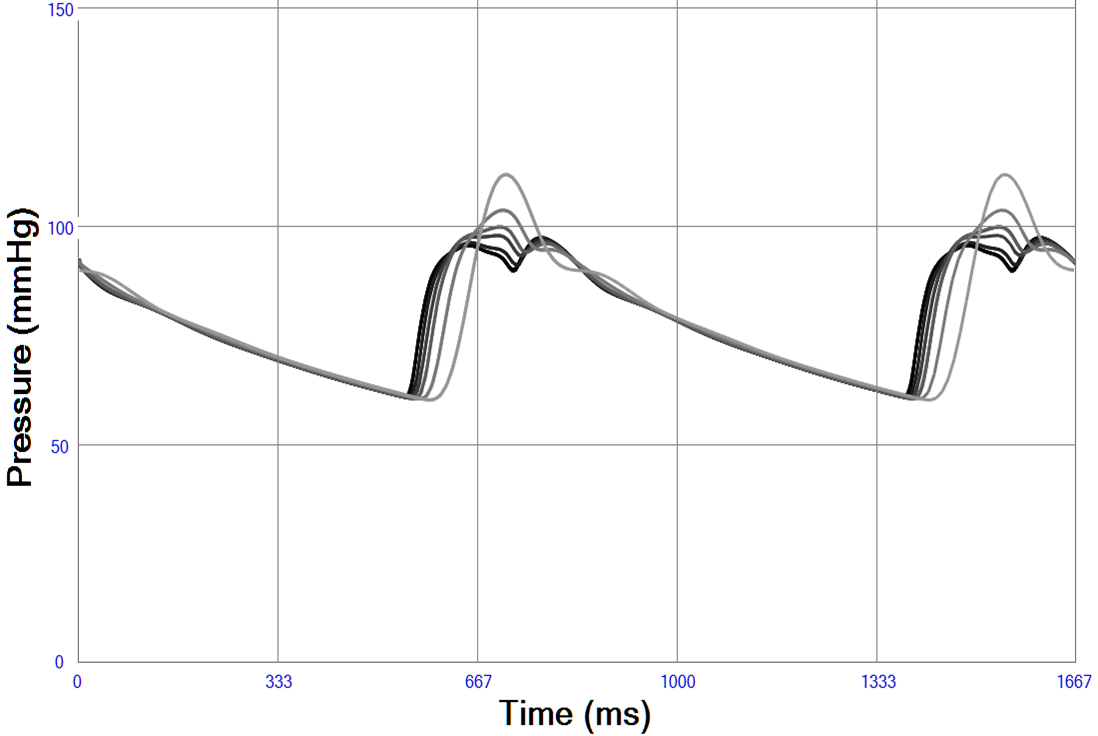

Supplement: Additional file 11: Figure S9 — Pressure changes simulated during two heart cycles in the aortic root (black), ascending aorta, proximal and distal aortic arch, descending aorta and a peripheral artery (light gray). The segments between the aortic root and the peripheral artery are shown in different shades of gray. [file 1475-925X-12-69-S11.tiff]

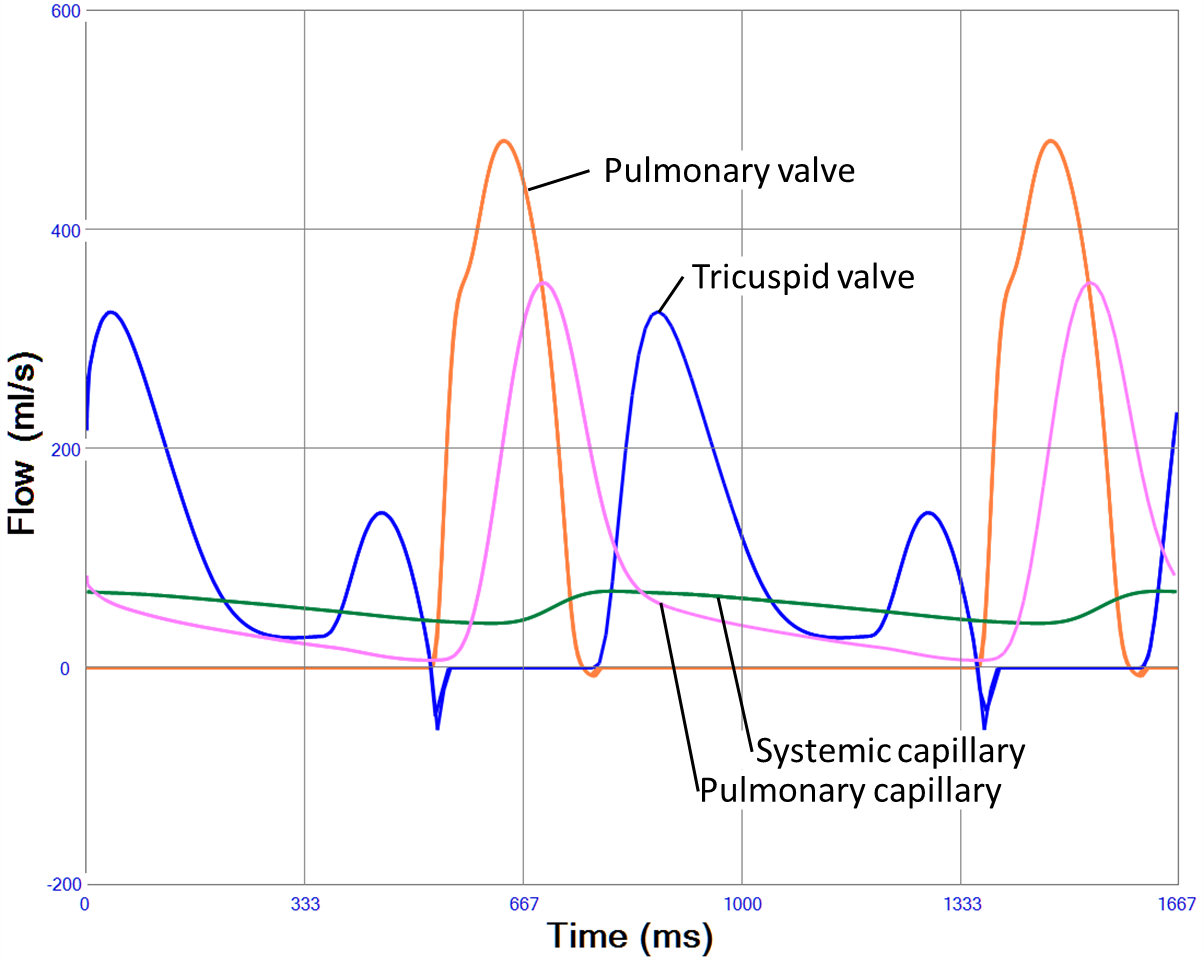

Supplement: Additional file 12: Figure S10 — Flow pulsatility in pulmonary capillaries (pink) is larger than in the systemic capillaries (green). Pulmonary valve flow (orange) and tricuspid valve flow (blue) resembling left-sided flows are also shown. [file 1475-925X-12-69-S12.tiff]

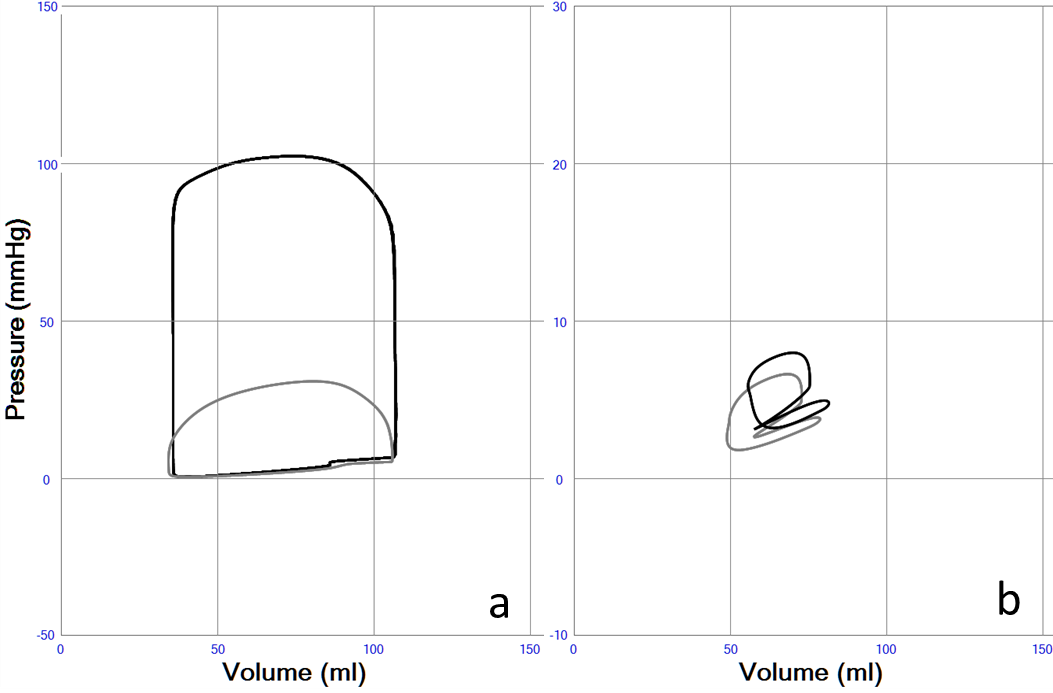

Supplement: Additional file 13: Figure S11 — a. Intracardiac pressure-volume loops from the left ventricle (black) and right ventricle (gray). b. Intracardiac pressure-volume loops from the left atrium (black) and right atrium (gray). [file 1475-925X-12-69-S13.tiff]

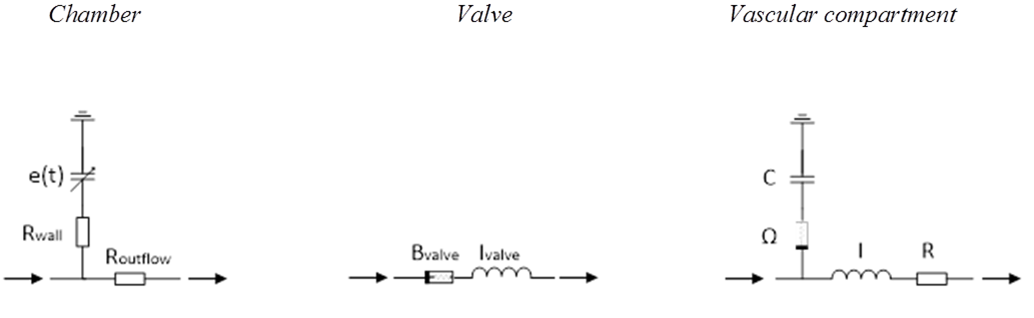

Supplement: Additional file 14: Figure S2 — Electrical analogue sketches showing details of the heart chambers, valves and vascular compartments. The arrows show directions of blood flow. e(t); time-varying elastance representing systolic and diastolic chamber properties, Rwall; a resistance representing viscous chamber wall properties, Routflow; a linear chamber outflow resistance, B; a non-linear Bernoulli valve resistance, Ivalve; an inductance representing flow inertia,, R; a linear resistance, I; an inductance representing flow inertia, C; a non-linear capacitor representing vascular elasticity, Ω; a non-linear resistance term representing viscous vascular wall properties. [file 1475-925X-12-69-S14.tiff]
