# Supplementary material for: Closed-loop real-time simulation model of hemodynamics and oxygen transport in the cardiovascular system
Source: Biomed Eng Online. 2013 Jul 10;12:69. doi: 10.1186/1475-925X-12-69 (PMC3751725; doi:10.1186/1475-925X-12-69)
Supplement: Additional file 2 — Closed-loop real-time simulation model of hemodynamics and oxygen transport in the cardiovascular system. Supplement. [file 1475-925X-12-69-S2.docx]

Closed-loop Real-time Simulation Model of Hemodynamics and Oxygen transport in the Cardiovascular System

SUPPLEMENT

**Supplement**

Detailed model properties are provided below. Additional file 1: Figure S1 shows an electrical analogue of the overall model, while Additional file 14: Figure S2 shows the details of the cardiac, valvular and vascular compartments in an electrical analogue sketch.

*The cardiac model*

The time-varying elastance function ***e(t)*** [11]describing each cardiac chamber contractile and passive properties is shown in Equation S1.

$$e\left( t \right)=e_{max}(v_{ed},q)\cdot a\cdot\left[ \frac{\left( \frac{t}{\alpha_{1}\cdot T} \right)^{n_{1}}}{1+\left( \frac{t}{\alpha_{1}\cdot T} \right)^{n_{1}}}\cdot\frac{1}{1+\left( \frac{t}{\alpha_{2}\cdot T} \right)^{n_{2}}} \right]+e_{min}\left( v \right)$$

*Equation S1.*

***T*** is cardiac cycle length. ***α_1_,*** ***n_1_***, ***α_2_*** and ***n_2_*** are dimensionless factors defining the shape of the chamber-specific elastance curves during contraction and relaxation phases (Additional file 3: Figure S3). The factor ***a*** is a normalizing scaling factor.

The systolic cardiac elastance contribution ***e_max_ (v_ed_, q)*** defines chamber contractility, as determined by the end-diastolic volume ***v***_ed_, output flow ***q***, the chamber-specific constants maximum elastance ***E_max_***, maximum end-diastolic volume ***V_ed,max_*** and maximum output flow ***Q_max_*** (Equation S2). This relation determines both the reduction of contractile force due to myocardial fiber stretch as determined by the quotient of end-diastolic volume ***v_ed_*** and maximum end-diastolic volume ***V_ed,max_*** according to the “heart law of Starling” in a similar way to Sun *et al* [8] (Figure 2) and the effect of an internal source resistance restricting maximum flow to ***Q_max_*** in each cardiac chamber in accordance with previous publications [11] making ventricular outflow more realistic.

$$e_{max}\left( v_{ed}, q \right)=E_{max}\cdot\left[ 1-\left( \frac{v_{ed}}{V_{ed,max}} \right)^{4} \right]\cdot\left[ 1-\frac{q}{Q_{max}} \right]$$

*Equation S2.*

The term ***e_min_(v)***  in Equation S1 is determined by a chamber-specific passive exponential pressure-volume relation defined by the minimum chamber elastance ***E_min_***, a constant ***σ*** and the zero volume ***v_0_*** as previously described [10] (Equation S3, Figure 2).

$$e_{\min}\left( v \right)=E_{min}\cdot e^{\sigma\cdot(v-v_{0})}$$

*Equation S3.*

The presence of the term ***e_min_(v)*** in Equation S1 both during systole and diastole, adds further realism during blood-volume overload and severely depressed ventricular function with cardiac dilatation.

Viscous properties of the cardiac chamber walls are simulated with a pressure-dependent resistor ***R_wall_*** [9] in series with the variable elastance/capacitance ***e(t)*** determining contractile properties (Additional file14: Figure S2).

*The heart valves*

Valve pressure gradients are composed of a Bernoulli resistance and an inertial term (Equation S4). The valve constants determining valve leaflet opening and closure speed is separate from the inertial parameter ***L*** determining blood flow inertia [11].

$$\Delta P=B\cdot q\cdot\left| q \right|+L\cdot\frac{dq}{dt}$$

*Equation S4.*

***L*** is calculated as described in the main text (Equation 1) to better describe inertia when valve area is changing. Bernoulli resistance ***B*** is determined by density ***ρ*** and squared valve area ***A*** and is the dominating term in creating the valvular pressure gradient (Equation S5, Additional file 6: Figure S6).

$$B=\frac{\rho}{2\cdot A^{2}}$$

*Equation S5.*

The valve area ***A*** used in the calculations above should be considered as an effective area, rather than the real area, but to simplify formulas they are considered identical [11]. Parameter values are found in Table S4.

*The pericardium*

A function (Equation S6, Additional file 7: Figure S7) relating intra-pericardial pressure ***p_pc_*** to total heart volume ***v_pc_*** is adopted from the literature [9]. Pericardial volume and stiffness can be modified and fluid added by volume to simulate pericardial restrictive properties and tamponade respectively. Total heart volume ***v_pc_*** includes the four heart chambers and the pericardial fluid volume, but excludes the myocardial volume, which is considered constant. The parameters ***p_pc,0_***, ***K_pc_***, ***V_pc,0_*** and ***φ_pc_*** are constants (Table S3).

$$p_{\mathrm{pc}}\left( v \right)=p_{pc,0}+K_{pc}\cdot e^{\frac{(v_{pc}-V_{pc,0})}{\varphi_{pc}}}$$

*Equation S6.*

As a further development of the previous model the minimal pericardial pressure ***p_pc,0_*** is allowed to be a negative value in agreement with what is found experimentally when measured during intrathoracic pressure changes or hypovolemia [16].

*The blood vessels*

An electrical analogue (Additional file 1: Figure S1 and Additional file 14: Figure S2) can be used to simplify the understanding of the fluid mechanical properties of each vascular segment as earlier described [9, 11]. All parameters and output of this vascular model can be expressed both in rheological unit equivalents and as properties of an electrical circuit. The individual vessel segments are modeled in the electrical analogue way with a non-linear resistance (***R***) in series with a non-linear inductor (***I***), a non-linear capacitance (***C***) and a non-linear resistance (***Ω***) in series with the capacitor. The parameter ***R*** corresponds to its counterpart with the same name in rheology, while ***I*** represents mass flow inertia and ***C*** the volume-dependent vascular compliance. The parameter ***Ω*** is the resistance for loading and unloading the capacitor in the electric analogue and represents viscous dampening of the pressure and flow pulse in rheology.

The volume constant ***V_0_*** is the volume and ***E_0_*** is the elastance at a normal mean pressure ***P_0_*** in each compartment (see table 9). The parameter ***φ*** (Equation S7) is a vessel-specific volume constant determining the non-linearity of the exponential pressure-volume relation.

Vascular elastance ***e_vascular_*** in each segment can be calculated by derivation of Equation S7 as seen in Equation S8. The relation describes an increase in elastance/stiffness with increasing volume corresponding to progressive distension of the vascular wall (Additional file 8: Figure S12). Compliance ***C*** is identical to inverted vascular elastance. Equation S9 is derived from Equation S8 and helpful when calculating ***φ*** from ***P_0_*** and ***E_0_***.

$$p\left( v \right)=P_{0}\cdot e^{\frac{v-V_{0}}{\varphi}}$$

*Equation S7.*

$$e_{\mathrm{vascular}}\left( v \right)=\frac{dp}{dv}=\frac{P_{0}}{\varphi}\cdot e^{\frac{v-V_{0}}{\varphi}}{=E}_{0}\cdot e^{\frac{v-V_{0}}{\varphi}}$$

*Equation S8.*

$$\varphi=\frac{P_{0}}{E_{0}}$$

*Equation S9.*

The vascular dimensions used as input parameters are shown in Table S5. Properties derived from these parameters are showed in Table S6-7.

*The coronary circulation*

Coronary circulation is simulated with a left and right coronary artery emptying in the right atrium. Flow is dependent on a fixed coronary vascular resistance ***R_coronary_***, aortic root pressure ***p_aortic root_***, and the maximum value of right atrial pressure ***p_ra_*** and intraventricular pressure ***p_ventricular_*** to create a vascular waterfall mechanism [29] resulting in realistic coronary flow (Equation S10, Additional file 9: Figure S8).

$$q_{coronary}=\left[ p_{aortic root}-maximum(p_{ventricular}, p_{ra}) \right]\cdot R_{coronary}$$

*Equation S10.*

*Baroreceptor reflex*

A baroreceptor reflex based on Sun *et al.* can be activated in the model that affects heart rate, cardiac contractility (maximum elastance) and arterial vascular resistance in a linear fashion with an adjustable gain (***G***) according to the example in Equation S11 that shows how cardiac cycle time ***T_new_*** is determined based on normal heart cycle time ***T_setpoint_*** , normal aortic pressure ***p_setpoint_*** and present aortic pressure ***p_aorta_***  [9] (Figure 9).

$$T_{new}=G_{HR}\cdot\left[ p_{aorta}-p_{setpoint} \right]+T_{setpoint}$$

*Equation S11.*

*Table S1. Basic cardiac parameters.*

|  | Contractility  ***E_max_*** | Time delay | Cycle time  ***T*** | ***α_1_*** | ***n_1_*** | ***α_2_*** | ***n_2_*** |
| --- | --- | --- | --- | --- | --- | --- | --- |
|  | *mmHg/ml* | *ms* | *ms* | *---* | *---* | *---* | *---* |
| Right atrium | 0.04 | 85 | 833 | 0.100 | 1.2 | 0.200 | 10 |
| Right ventricle | 0.60 | 245 | 833 | 0.200 | 1.2 | 0.250 | 10 |
| Left atrium | 0.05 | 95 | 833 | 0.100 | 1.2 | 0.200 | 10 |
| Left ventricle | 2.80 | 245 | 833 | 0.200 | 1.2 | 0.250 | 20 |

*Table S2. Basic diastolic and other cardiac parameters.*

|  | Stiffness  ***E_min_*** | Sigma  ***σ*** | Maximal volume  ***V_ed,max_*** | Maximal flow ***Q_max_*** | Viscous damping  ***R_wall_*** |
| --- | --- | --- | --- | --- | --- |
|  | *mmHg/ml* | *ml^-1^* | *ml* | *ml/s* | *mmHg·s/ml* |
| Right atrium | 0.06 | 0.0015 | 400 | 4000 | 0.00050 |
| Right ventricle | 0.04 | 0.0030 | 400 | 2000 | 0.00020 |
| Left atrium | 0.07 | 0.0015 | 400 | 4000 | 0.00050 |
| Left ventricle | 0.05 | 0.0030 | 400 | 2000 | 0.00005 |

*Table S3. Pericardial and septal parameters.*

| Minimum pericardial pressure  ***p_pc,0_*** | Pericardial pressure constant ***K_pc_*** | Pericardial volume constant ***V_pc,0_*** | Pericardial volume constant ***φ_pc_*** | Atrial septal stiffness ***E_sa0_*** | Ventricular septal stiffness ***E_sv0_*** |
| --- | --- | --- | --- | --- | --- |
| *mmHg* | *ml/s* | *ml* | *ml* | *mmHg/ml* | *mmHg/ml* |
| -2 | 1.0 | 320 | 50 | 12 | 46 |

*Table S4. Valve parameters.*

|  | Closed area | Open area | Bernoulli resistance | Opening constant | Closure constant |
| --- | --- | --- | --- | --- | --- |
|  | *cm^2^* | *cm^2^* | *mmHg·s/ml* | *(mmHg·s)^-1^* | *(mmHg·s) ^-1^* |
| Tricuspid valve | 0.0 | 5.0 | 0.00040 | 40 | 30 |
| Pulmonary valve | 0.0 | 5.0 | 0.00040 | 30 | 30 |
| Mitral valve | 0.0 | 5.0 | 0.00040 | 40 | 20 |
| Aortic valve | 0.0 | 5.0 | 0.00040 | 20 | 20 |

*Table S5. Vascular parameters – dimensions – input data.*

|  | Length | Radius | Thickness | Young’s modulus | Number | Normal mean pressure |
| --- | --- | --- | --- | --- | --- | --- |
|  | l | r | h | Y | n | P_0_ |
|  | *cm* | *cm* | *cm* | *mmHg* | *---* | *mmHg* |
| Aortic root | 2,00 | 1,6000 | 0,16300 | 3000 | 1 | 80 |
| Ascending aorta | 4,00 | 1,4700 | 0,16300 | 3000 | 1 | 80 |
| Proximal aortic arch | 2,00 | 1,2600 | 0,12600 | 3000 | 1 | 80 |
| Distal aortic arch | 3,90 | 1,1900 | 0,11500 | 3000 | 1 | 80 |
| Descending aorta | 20,00 | 1,1000 | 0,10000 | 3000 | 1 | 80 |
| Peripheral arteries | 30,00 | 0,1600 | 0,05000 | 3000 | 80 | 80 |
| Resistance arteries | 4,00 | 0,0250 | 0,01000 | 3000 | 7000 | 80 |
|  |  |  |  |  |  |  |
| Right carotid artery | 20,00 | 0,4730 | 0,06300 | 3000 | 1 | 80 |
| Right carotid resistance arteries | 4,00 | 0,0250 | 0,02000 | 3000 | 1200 | 80 |
| Right carotid capillaries | 0,02 | 0,0005 | 0,00001 | 3000 | 2400000000 | 6 |
| Right carotid vein | 12,00 | 0,1000 | 0,00100 | 3000 | 1500 | 4 |
|  |  |  |  |  |  |  |
| Left carotid artery | 20,00 | 0,4130 | 0,06300 | 3000 | 1 | 80 |
| Left carotid resistance arteries | 4,00 | 0,0250 | 0,02000 | 3000 | 1200 | 80 |
| Left carotid capillaries | 0,02 | 0,0005 | 0,00001 | 3000 | 2400000000 | 6 |
| Left carotid vein | 12,00 | 0,1000 | 0,00100 | 3000 | 1500 | 4 |
|  |  |  |  |  |  |  |
| Systemic capillaries | 0,02 | 0,0005 | 0,00001 | 3000 | 11200000000 | 6 |
| Capacitance vessels | 12,00 | 0,1000 | 0,00100 | 3000 | 7000 | 4 |
| Superior caval vein | 12,00 | 1,4000 | 0,15000 | 3000 | 1 | 4 |
| Inferior caval vein | 20,00 | 1,4000 | 0,15000 | 3000 | 1 | 4 |
|  |  |  |  |  |  |  |
| Pulmonary artery | 10,00 | 1,5000 | 0,12000 | 3000 | 1 | 12 |
| Pulmonary resistance arteries | 1,00 | 0,0600 | 0,01000 | 3000 | 2000 | 12 |
| Pulmonary capillaries | 0,02 | 0,0005 | 0,00001 | 3000 | 8000000000 | 10 |
| Pulmonary small veins | 6,00 | 0,0800 | 0,00100 | 3000 | 3000 | 8 |
| Pulmonary veins | 6,00 | 0,7000 | 0,10000 | 3000 | 4 | 6 |

*Table S6. Vascular parameters – calculated from values in table S5.*

|  | Volume | Volume | Elastance | Resistance | Inductance |
| --- | --- | --- | --- | --- | --- |
|  | Vₒ | φ | Eₒ | Rₒ | Lₒ |
|  | *ml* | *ml* | *mmHg/ml* | *mmHg·s/ml* | *mmHg·s^2^/ml* |
| Aortic root | 16.08 | 8.42 | 9.50034 | 0.00019 | 0.00019910 |
| Ascending aorta | 27.15 | 13.06 | 6.12515 | 0.00053 | 0.00047108 |
| Proximal aortic arch | 9.98 | 5.32 | 15.03732 | 0.00049 | 0.00032115 |
| Distal aortic arch | 17.35 | 9.58 | 8.35475 | 0.00120 | 0.00070170 |
| Descending aorta | 38.01 | 22.30 | 3.58726 | 0.00421 | 0.00210095 |
| Peripheral arteries | 193.02 | 32.94 | 2.42851 | 0.35053 | 0.00371177 |
| Resistance arteries | 54.98 | 7.33 | 10.91348 | 0.96403 | 0.00024028 |
|  |  |  |  |  |  |
| Right carotid artery | 14.06 | 5.63 | 14.21244 | 0.24415 | 0.02262250 |
| Right carotid resistance arteries | 9.42 | 0.63 | 127.32394 | 5.25036 | 0.00135436 |
| Right carotid capillaries | 37.70 | 7.54 | 0.79577 | 0.08381 | 0.00000001 |
| Right carotid vein | 565.49 | 150.80 | 0.02653 | 0.04674 | 0.00019797 |
|  |  |  |  |  |  |
| Left carotid artery | 10.72 | 3.75 | 21.35019 | 0.42048 | 0.02968836 |
| Left carotid resistance arteries | 9.42 | 0.63 | 127.32394 | 5.26930 | 0.00135680 |
| Left carotid capillaries | 37.70 | 7.54 | 0.79577 | 0.08413 | 0.00000001 |
| Left carotid vein | 565.49 | 150.80 | 0.02653 | 0.04685 | 0.00019821 |
|  |  |  |  |  |  |
| Systemic capillaries | 175.93 | 35.19 | 0.17052 | 0.01797 | 0.00000000 |
| Capacitance vessels | 2 638.94 | 703.72 | 0.00568 | 0.00991 | 0.00004219 |
| Superior caval vein | 73.89 | 1.84 | 2.17504 | 0.00194 | 0.00156017 |
| Inferior caval vein | 123.15 | 3.07 | 1.30502 | 0.00320 | 0.00259118 |
|  |  |  |  |  |  |
| Pulmonary artery | 70.69 | 7.07 | 1.69765 | 0.00128 | 0.00115793 |
| Pulmonary resistance arteries | 22.62 | 1.09 | 11.05243 | 0.02426 | 0.00003566 |
| Pulmonary capillaries | 125.66 | 41.89 | 0.23873 | 0.02674 | 0.00000000 |
| Pulmonary small veins | 361.91 | 154.42 | 0.05181 | 0.03046 | 0.00007991 |
| Pulmonary veins | 36.95 | 1.03 | 5.80011 | 0.00393 | 0.00078567 |
|  |  |  |  |  |  |
| Right coronary artery | - | - | - | 80.00 | - |
| Left coronary artery | - | - | - | 40.00 | - |

*Table S7. More properties of the vascular system – calculated from values in table S5.*

|  | Volume | Vascular surface area | Vascular wall volume |
| --- | --- | --- | --- |
|  | *ml* | *m^2^* | *ml* |
| Systemic arteries | 438 | 0.87 | 230 |
| Systemic capillaries | 251 | 101 | 10 |
| Systemic veins | 3967 | 7.57 | 118 |
| Pulmonary arteries | 93 | 0.08 | 19 |
| Pulmonary capillaries | 126 | 50.3 | 5 |
| Pulmonary veins | 399 | 0.92 | 20 |
| **TOTAL** | **5274** | **160** | **401** |
