# Supplementary material for: Closed-loop real-time simulation model of hemodynamics and oxygen transport in the cardiovascular system
Source: Biomed Eng Online. 2013 Jul 10;12:69. doi: 10.1186/1475-925X-12-69 (PMC3751725; doi:10.1186/1475-925X-12-69)
Supplement: Additional file 10 — Sensitivity analysis. [file 1475-925X-12-69-S10.doc]

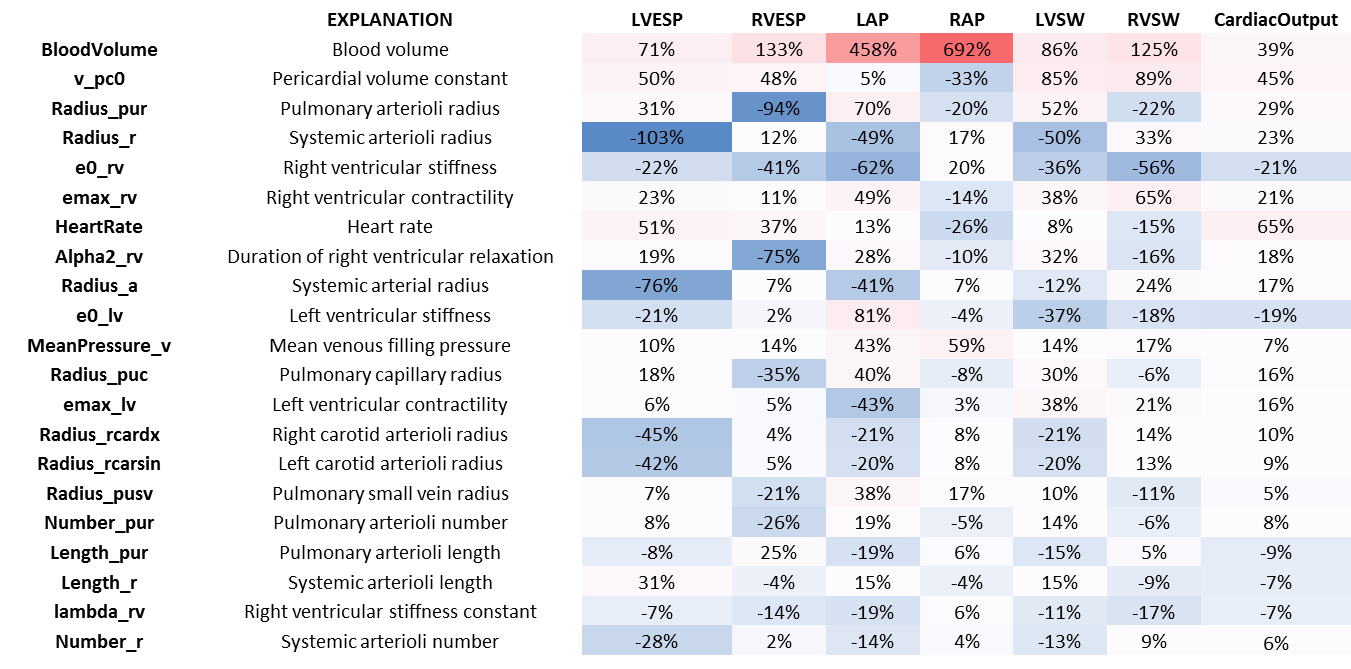


All parameters with a mean sensitivity above 10% are presented sorted according to their mean effect on seven parameters (*LVESP* left ventricular end-systolic pressure, *RVESP* right ventricular end-systolic pressure, *LAP* left atrial pressure, *RAP* right atrial pressure, *LVSW* left ventricular stroke work, *RVSW* right ventricular stroke work and Cardiac Output ). Effects are evaluated after 60 seconds (=steady-state) after a 10% increase in the parameter (changed one by one). Each cell background color shows effect magnitude on a color scale, where red implies that an increase in input produces an increase in output, whereas blue implies that an increase in input produces a decrease in output, while shaded background colors are in between.
